# Text message reminders for adolescents with poorly controlled type 1 diabetes: A randomized controlled trial

**DOI:** 10.1371/journal.pone.0248549

**Published:** 2021-03-15

**Authors:** Nour Ibrahim, Jean-Marc Treluyer, Nelly Briand, Cécile Godot, Michel Polak, Jacques Beltrand

**Affiliations:** 1 Clinical Research Unit, Paris Descartes, *Assistance Publique-Hôpitaux de Paris*, Necker University Hospital, Paris, France; 2 French Clinical Research Group in Adolescent Medicine and Health, Paris, France; 3 Pharmacology and Drug Evaluation in Children and Pregnant Women EA7323, Paris Descartes University, Sorbonne Paris Cité, Paris, France; 4 Pediatric Endocrinology, Gynecology, and Diabetology Department, *Assistance Publique-Hôpitaux de Paris*, Necker University Hospital, Paris, France; Weill Cornell Medical College in Qatar, QATAR

## Abstract

**Background:**

Among adolescents with type 1 diabetes, some experience great difficulties with treatment adherence, putting them at high risk of complications. We assessed the effect of text messaging (Short Messaging Service [SMS]) on glycemic control.

**Methods:**

A two-arm open label randomized controlled trial enrolled adolescents with type 1 diabetes aged 12–21 years with baseline HbA1c ≥ 69 mmol/mol (8.5%). The intervention group received daily SMS reminders at self-selected times about insulin injections while the control group received standard of care. The patients allocated to the control group were not aware of the intervention.

**Results:**

92 patients were randomized, 45 in the SMS arm and 47 in the control arm. After 6 months, median HbA1c level was significantly lower in the intervention arm: 73 mmol/mol (8.8%) in the SMS arm and 83 mmol/mol (9.7%) in the control arm in the intent-to-treat analysis (*P* = 0.03) but no longer in the per protocol analysis (P = 0.65). When we consider the proportions of patients whose HbA1c level decreased by at least 1% between baseline and 6 months, we find a significant difference among patients whose baseline HbA1c was ≥ 80 mmol/mol (9.5%) (n = 56): 60% in the SMS arm and 30.6% in the control arm had lowered their HbA1c level (*P* = 0.03) in the intent-to-treat analysis but not in the per-protocol analysis (P = 0.50). Patients in the SMS arm reported high satisfaction with the intervention.

**Conclusions:**

While there is a trend to lower HbA1c in the intervention group, no firm conclusions can yet be drawn. Further studies are needed to address methodological issues as we believe these interventions can support behavior change among adolescents with poorly controlled type 1 diabetes. ClinicalTrials.gov identifier: NCT02230137.

## Introduction

Good control of type 1 diabetes can only be obtained via a complex care regimen that is largely administered by the patients themselves and requires a high level of self-discipline [1], which may raise considerable challenges for adolescents [2–4]. In France, adolescents with type 1 diabetes receive follow-up at tertiary-care hospitals, which provide them with a broad range of medical, psychological, and supportive interventions [4]. Despite this comprehensive care, poor glycemic control is still a reality, exposing these young patients to chronic diabetic complications [5, 6]. One reason for poor control in adolescents is failure to adhere to the stringent insulin injection regimen [7, 8]. And yet, we as clinicians have very little means to effect change in behavior in adolescents with poorly controlled type 1 diabetes. Given the increasing worldwide prevalence of type 1 diabetes [9, 10], pratical, low-cost interventions which can improve adherence and clinical outcomes in this population would be valuable.

Most adolescents and young adults own a mobile phone. A study in adolescents with diabetes showed that text messaging via the Short Messaging Service (SMS) was their preferred means of communication [11]. Mobile phone messaging can be a low-cost approach to support patients between clinic visits. However, whether sending SMSs to improve treatment adherence results in better glycemic control is unclear [12–14]. Most of the published available studies to date were not randomized and had no control group [14–17]. Many of them were preliminary pilot studies with limited sample sizes [14, 17–21], and some included and some included multiple interventions including SMS, making the specific impact of SMSs impossible to determine [19, 22]. Finally, very few studies focused specifically on patients with poor glycemic control [15, 21, 23–25].

The primary objective of this randomized controlled study was to evaluate whether daily SMS reminders about insulin injections improved glycemic control in adolescents and young adults with poorly controlled type 1 diabetes. The secondary objectives were to compare quality of life in the two groups and to assess patient satisfaction with the intervention.

## Materials and methods

### Ethical considerations

The study was approved by our institutional review board (Comité de Protection des Personnes Ile de France II, N° ID RCB: 2014-A00915-42) and registered on ClinicalTrials.gov (#NCT02230137; Text Messages for Adolescents with Poorly Controlled Type 1 Diabetes). The protocol was amended twice after trial initiation. The first amendment added a satisfaction survey at the end of the trial among patients in the intervention group. The second extended the age of eligibility for the trial from 12–18 years to 12–21 years, due to slow recruitment. Full trial protocol is available among S1 File.

After the patients had been approached to enroll in this cohort, non-objection by the parents and by adolescents to the text messaging intervention were documented in each patient’s medical record (date on which the information was provided, objection expressed or not, and signature of the person in charge of the visit).

As our study is a research work with minimal risks (standard care was not modified by this study), only non-objection by the parents and by adolescents were obtained. No written consent was obtained. The ethics committee approved this consent procedure.

### Study design

Owing to the nature of the intervention, patients cannot be blinded to the allocation group. Therefore, we performed a 6-month single-consent Zelen randomized clinical trial with two parallel arms [26]. In a Zelen design, all participants are asked to enroll in a cohort. Then, participants are randomized prior to seeking consent for the intervention. Only participants allocated to the intervention group are then approached and offered the intervention. In this way, patients randomized to the control group are unaware of the existence of the intervention. This design reduced bias by avoiding the feeling of disappointment among patients randomized to the control group. Furthermore, it could increase recruitment as it promoted participation and was less time consuming for the clinicians.

More specifically, in our study, all eligible patients were approached and asked to provide consent to enroll in a cohort by their diabetologist during scheduled clinic visits. Non-objection was obtained from the patients in every case and, for patients younger than 18 years, from the parents also. Those who consented were then randomized online by the first author (NI) to the control group (standard treatment alone) or to the SMS group (standard treatment with SMS insulin injection reminders) using a computer generated random number list prepared by the data manager. Random allocation was in a 1:1 ratio, with no restrictions. Only the patients allocated to the SMS group had an extra 10-minute interview during which the intervention was explained to them and were then asked for consent to the intervention. They started receiving SMS within one week. Since the patients used to leave after their clinic visit, they had scarce opportunity to interface with each other. There were no activities offered by the hospital for young diabetics during the trial.

### Subjects

Patients were recruited at the endocrinology department of the Necker Children’s Hospital, Paris, France, between February and October 2015. Inclusion criteria were age 12 to 21 years, type 1 diabetes diagnosed at least 6 months earlier with an HbA1c level at study inclusion ≥ 69 mmol/mol (8.5%), owning a personal cell phone, being fluent in French, not having being diagnosed for an acute psychiatric disease, not being pregnant and being willing to attend two follow-up visits, after 3 and 6 months.

### Data collection

At baseline, after the patient had consented to enrolment in a cohort, demographic data and information on the history of type 1 diabetes and any other health issues were collected from the electronic medical records. Ethnicity was not available. Body mass index was not collected for this study.

The primary outcome measure was the HbA1c level 6 months after study inclusion. HbA1c was measured at baseline then 3 and 6 months later.

Quality of life was assessed by asking the patients to complete the French version of the PedsQL 4.0 at baseline then 6 months later [27, 28]. In the SMS group, patient satisfaction with the intervention was evaluated at the end of the trial using a 7-item questionnaire specifically developed for the study.

Adverse events were recorded. Severe hypoglycemia was defined as loss of consciousness and/or seizure and/or any episode that required assistance from another person to allow the patient to recover. Hypogycemia was defined as blood glucose < 60 mg/dl or < 3.3 mmol/l. Diabetic ketoacidosis was defined as serum pH <7.2 combined with ketonuria, hyperglycemia, and clinical symptoms.

### Intervention

This programme was designed by the authors and validated by the research team of the Clinical Research Unit of Necker Enfants Malades University Hospital. We created the Diabeto-SMS program designed to help patients remember their insulin injections. The SMSs sent by the program focus solely on insulin injections. No other messages were transmitted about diabetes at the same time, there was no messages concerning the blood glucose measurements. We did not ask our patients if they had any diabetes apps on their mobile phone or if they were using continuous glucose monitor (CGM). CGM was not very widespread at the time of the study (2014–2015) as its cost was not covered by French social security until June 2017. The patients could choose the number of messages per day (one to four), their timing, and the insulin type specified in the message (long-acting insulin or short-acting insulin analogs). There was no dose reminder. In pump patients, the messages prompted only bolus injections. The messages were tailored to each patient’s daily schedule and treatment regimen so that they were sent before mealtimes and before bedtime (“Hello, you’re about to have your meal, consider doing your short-acting insulin injection” or “Hello, you’re about to have your dinner, consider doing your long-acting insulin injection”). Data on each patient were entered into the program, which then sent the messages automatically via an Internet platform. Patients could stop receiving the messages by texting the word “stop” and they could reallow receiving them later if they wanted to.

### Control group

Patients randomized to the control group had consultation and HbA1c blood test with their diabetologist every 3 months as per standard of care. If needed, they could receive counselling from a specialized nurse, dietetician and psychologist. For this study, their diabetologist and the first author (NI) informed them that anonymous data would be collected from their electronic medical records, including HbA1c level at baseline and during 6 months. Quality of life was assessed during clinic visits.

### Statistical analysis

Based on results from Hvidore study [29], we assumed that HbA1c was normally distributed, with a standard deviation at 1.6. Our study was designed to detect an at least 1% difference in HbA1c values between the two groups after 6 months, with a two-sided alpha risk equal to 5% and 80% power, 41 patients randomized per group were required. We anticipated a 10% rate of crossover between groups and therefore increased the estimated sample size by 20%. Thus, 100 patients, 50 per group, were needed.

Statistical tests were two-sided and P values <0.05 were considered statistically significant. Baseline characteristics of the two groups were described as median (range) for quantitative variables and frequencies (%) for qualitative variables. Our intent-to-treat (ITT) population consisted of all randomized participants. A per-protocol population was also defined, including all randomized patients with evaluable primary outcome, after re-classification according to the current intervention: patients randomized in the SMS group who did not receive the intervention were classified in the control arm.

The primary outcome, i.e., the median HbA1c level after 6 months, was compared between the two groups by applying the Wilcoxon test. Missing 6-month HbA1c data were replaced by the baseline HbA1c data. Median difference was calculated following Hodges-Lehmann estimate.

Despite randomization, we were unfortunate to find an HbA1c difference at baseline. Hba1c was not normally distributed in our study. To mitigate this baseline HbA1c difference, we added a post-hoc analysis comparing the proportion of patients whose HbA1c level decreased by at least 1% between baseline and 6 months. Thus, the proportions of patients whose HbA1c level decreased by at least 1% between baseline and 6 months were compared using a Wilcoxon test. Patients with missing HbA1c data at 6 months were classified as not having an at least 1% decrease in their HbA1c level versus baseline.

We performed an intent-to-treat analysis followed by a per-protocol analysis. Finally, we repeated these analyses in the subgroup of patients whose HbA1c level was ≥ 80 mmol/l (9.5%) at baseline, which was not pre-planned.

PedsQl 4.0 scores after 6 months were compared between the two groups using chi-square test (or Fisher’s exact test when it was appropriate).

The results of the satisfaction survey were described as n (%) of patients satisfied with each item.

Analyses were conducted using SAS statistical software version 9.1 (SAS, Institute Inc., Cary, NC).

## Results

### Participants

**Fig 1:**
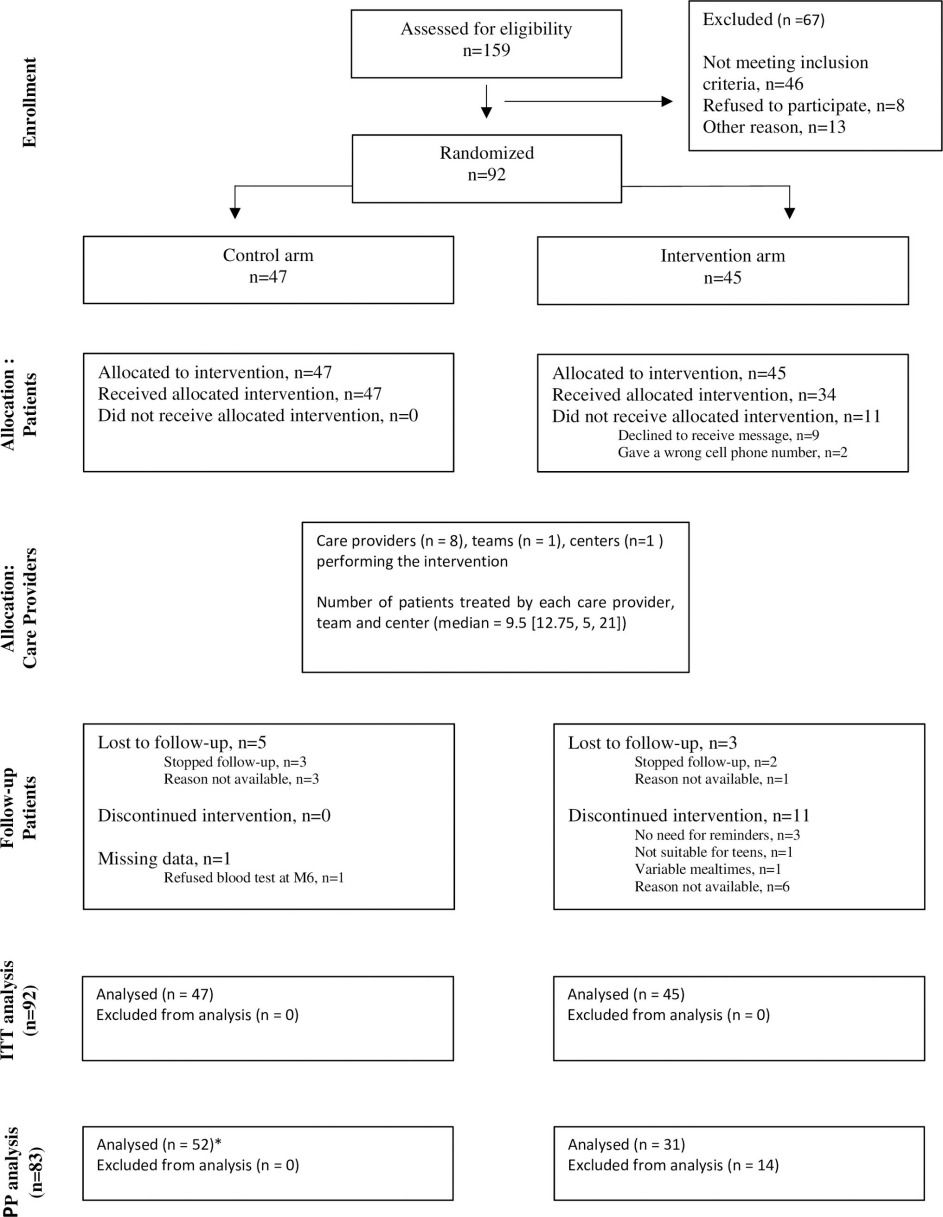
Patient flow chart. *In the per protocol analysis, unexposed patients were the control patients, and the patients randomized to the intervention who refused to receive the SMSs or gave a wrong cell phone number. Exposed patients were the patients randomized to the intervention who received SMSs.

Fig 1 shows the progress of the patients through the trial, according to the Consort flow diagram. Of the 159 patients initially identified as eligible for participation based on their medical records, 92 were included. Among them, 45 were allocated to the SMS and 47 to the control group. Finally, 42 SMS and 42 no SMS patients completed the study, yielding an 8.6% attrition rate. End of follow-up occurred in May 2016.

Table 1 reports the baseline characteristics in the overall population. Significant differences occurred for insulin pump use (33% in the SMS arm and 19% in the No SMS arm) and baseline median HbA1c level: 77 mmol/mol (9.2%) in the SMS arm and 87 mmol/mol (10.1%) in the control arm. When considering the patients whose HbA1c level was ≥ 80 mmol/mol (9.5%) at baseline (n = 56 patients), the same difference occurred for insulin pump use but not for baseline median HbA1c level as there was a smaller difference: 88 mmol/mol (10.15%) in the SMS arm and 92 mmol/mol (10.6%) in the control arm.

**Table 1:** Baseline characteristics of the study participants according to group.

Variable	Overall population	Control arm	SMS arm
	n = 92	n = 47	n = 45
Median age, years [IQR]	14.8 [13.6;16.5]	14.6 [13.2;15.9]	15.4[14.0;16.7]
Females, n (%)	62.0	63.8	60.0
Median duration of diabetes, years [IQR]	8.0 [5.1;11.4]	6.8 [5.0;11.4]	8.7 [6.0;11.4]
Insulin pump, n (%)	26.1	19.1	33.3
HbA1c level, (mmol/l)	83	87	77
Median HbA1c level, (%) [IQR]	9.7 [9.1;10.8]	10.1 [9.5;11.0]	9.2 [8.9;10.1]
Median PedsQL 4.0 score† [IQR]	83 [74;89]	82 [76;88]	83 [74;91]

IQR: interquartile range.

**†**The highest possible value of the PedsQL 4.0 score is 100.

### Use of the SMS system

Of the 45 patients allocated to the SMS group, 9 (20%) did not consent to the intervention. One patient withdrew from the study on the 3-month visit.

The system sent out a total of 14 000 SMSs to the remaining 36 SMS patients. Patients scheduled a median of 3 SMSs per day. Each day, 4 participants received 1 SMS, 10 received 2 SMSs, 14 received 3 SMSs, and 8 received 4 SMSs. Median number of days receiving SMSs was 181 [110;182.5]. Of the 36 patients, 11 stopped the messages before the end of the trial, after a median of 85 days [18;109]. Reasons for stopping the messages were available for 5 patients and included no need for insulin injection reminders (n = 3), variable mealtimes (n = 1), and a comment that the text messages were not suitable for teens (n = 1). Those 11 patients who stopped the messages were kept in the intervention group in the per protocol analysis as they had partial exposure to the intervention during the follow-up period.

In 4 patients, the messages were identified by the mobile network operator as advertisements and were therefore stopped, before being restored 2 weeks later. Two patients had given a wrong mobile phone number.

### Glycemic control at 3 months

Median Hba1c was not significantly lower in the intervention arm at 3 months: 80 mmol/mol (9,5%) in the SMS arm and 77 mmol/mol (9,2%) in the control arm, in the intention-to-treat analysis (p = 0.18).

### Glycemic control at 6 months

The median HbA1c level after 6 months was significantly lower in the SMS arm than in the no SMS arm in the intent-to-treat analysis with a significant size effect (Hodge-lehman estimate = 0.7%; 95% confidence interval, 0.1–1.3).

In the per-protocol analysis, the median HbA1c level was lower in the SMS group but the difference was no longer significant. Among the 45 patients in the SMS arm, the 11 patients who did not receive the intervention (9 declined to participate et 2 gave wrong cell numbers) and the 3 patients with missing data were excluded for the per protocol analysis. Among the 47 patients in the control arm for the per protocol analysis, the 6 patients with missing data were excluded from this analysis. In addition, the 11 patients randomized in the SMS group but who did not receive the intervention were classified in the control arm. Results are shown in Table 2.

**Table 2:** Median HbA1c level at 6 months.

Diabéto-SMS results	Control arm	SMS arm	*P* value*
*ITT* †	(n = 47)	(n = 45)	
Median HbA1c level, (mmol/mol) [IQR]	83 [69–96]	73 [67–81]	
Median HbA1c level, (%) [IQR]	9.7 [8.5–10.9]	8.8 [8.3–9.6]	0.03*
*PP*‡	(n = 52)	(n = 31)	
Median HbA1c level, (mmol/mol) [IQR]	81 [67–91]	73 [68–84]	
Median HbA1c level, (%) [IQR]	9.2 [8.3–10.5]	8.8 [8.4–9.8]	0.65*

**†** ITT: intent-to-treat analysis.

**‡** PP: per-protocol analysis.

*Wilcoxon test.

To take into account median HbA1c difference at baseline we compared the proportions of patients whose HbA1c level decreased by at least 1% between baseline and 6 months. When considering the whole population, the proportion of patients whose HbA1c level decreased by at least 1% from baseline to the 6-month time point was not significantly different between the two groups.

When considering the patients whose HbA1c level was ≥ 80 mmol/mol (9.5%) at baseline (n = 56 patients), the proportion of patients whose HbA1c level decreased by at least 1% between baseline and 6 months was significantly higher in the SMS group in the intent-to-treat analysis but not in the per-protocol analysis. There were 6 missing data in the control arm and 3 missing data in the SMS arm. The 11 patients in the SMS arm who did not receive the intervention (9 declined to participate et 2 gave wrong cell numbers) were classified in the control arm. Results are shown in Table 3.

**Table 3:** Number of patients whose HbA1c level decreased by at least 1% between baseline and 6 months.

Whole population	Control arm	SMS+ arm	*P* value
ITT †	(n = 47)	(n = 45)	
n (%)	11 (23.4)	13 (28.9)	0.55*
*PP* ‡	(n = 52)	(n = 31)	
n (%)	16 (30.8)	8 (25.8)	0.63*
Subset of patient whose baseline HbA1C > 80 mmol/mol			
ITT †	(n = 36)	(n = 20)	
n (%)	11 (30,6)	12 (60)	0.03*
*PP* ‡	(n = 35)	(n = 15)	
n (%)	15 (42,9)	8 (53,3)	0.50*

**†** ITT: intent-to-treat analysis.

**‡** PP: per-protocol analysis.

* Chi-square test.

### Quality of life

The PedsQL 4.0 score was not significantly different between the two groups: median PedsQL 4.0 score was 83 in the control arm and 86 in the SMS arm (p = 0.2).

### Patient satisfaction

**Fig 2:**
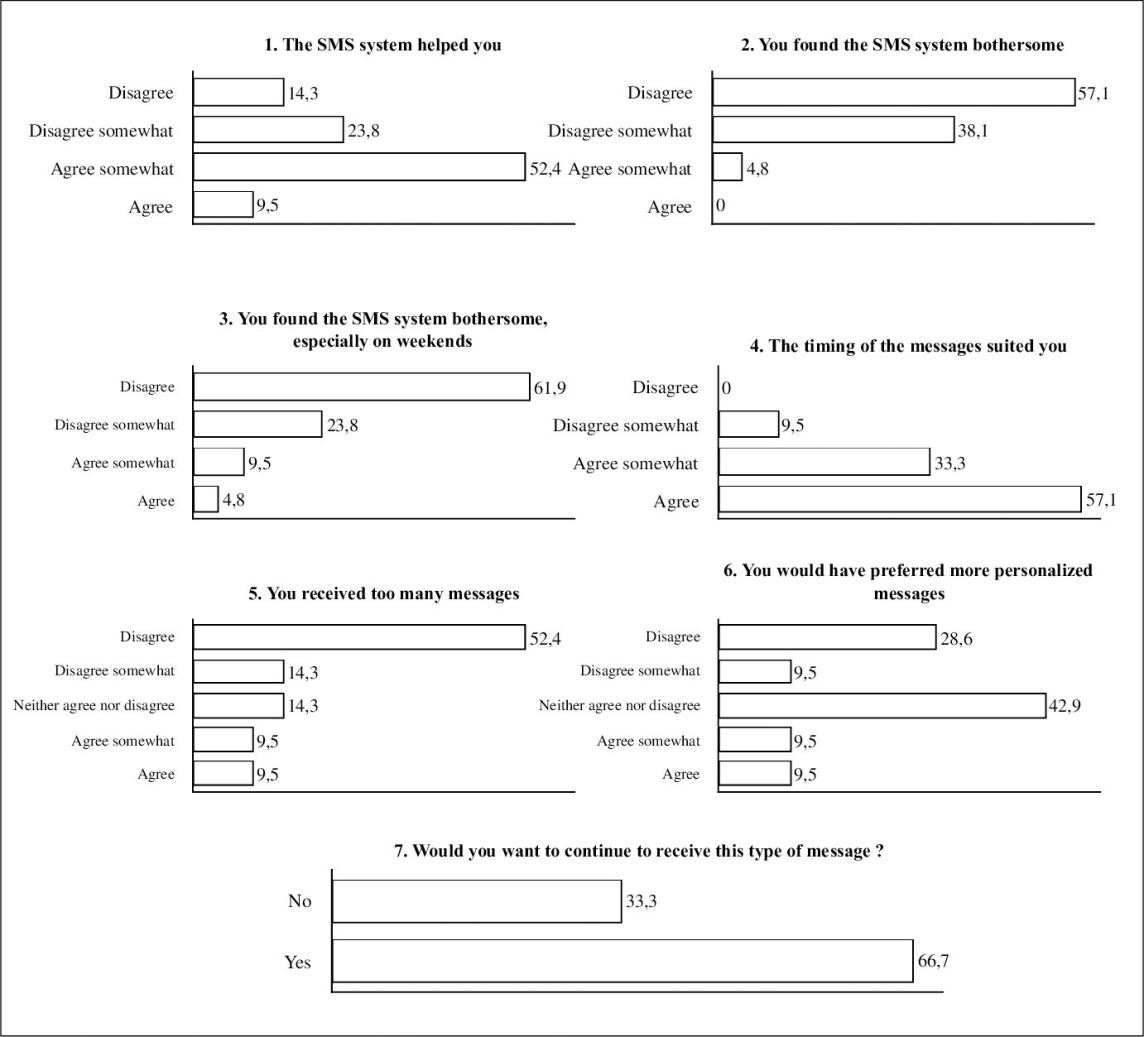
Satisfaction questionnaire completed by 21 patients randomized in the intervention arm (SMS arm). The data are percentages.

Of the 45 patients allocated to the intervention, 21 (47%) completed the satisfaction questionnaire. Among them, 13 (61.2%) felt that the intervention helped them and 14 (66.7%) wanted to continue to receive the messages (Fig 2). Response rate was restricted because diabetologists often forgot to submit this survey at the end of the study because of insufficient visit time.

### Adverse events

In each group, 1 patient experienced a single episode of severe hypoglycemia. Ketoacidosis occurred in 1 SMS patient and 3 control patients.

## Discussion

This study focused on the use of automated cell-phone text messages (SMSs) sent as reminders about insulin injections to a patient population made up exclusively of adolescents with poorly controlled type 1 diabetes. Among the overall population of patients receiving messages, glycemic control improved significantly in the intent-to-treat-analysis, yet the two groups differed regarding baseline HbA1c level. We therefore compared the two groups for the proportions of patients whose HbA1c level decreased by at least 1% between baseline and 6 months. Glycemic control was significantly improved only within the patient subset with baseline HbA1c levels ≥ 80 mmol/mol (9.5%) receiving messages in the intent-to-treat analysis but no longer in the per-protocol analysis. Thus, no firm conclusions can be drawn from our study regarding glycemic control, which is consistent with some of the mixed results reported in the literature [12, 30–32] We elected to carry out an a pragmatic low-cost intervention study design. It was easy to set up given the simple nature of the insulin injection reminders, as well as very inexpensive and marginally time-consuming for medical personnel. The system was entirely automated. We focused on the use of SMSs because it is the preferred mode of communication for adolescents. Among patients randomized to the intervention, 80% were willing to receive text messages, confirming the good acceptability of this method [12, 21, 22]. Furthermore, two-thirds of the patients in the SMS arm who completed the satisfaction questionnaire wanted to continue to receive the messages. Interestingly, the SMSs were well-accepted by patients who used an insulin pump on which they could pre-program insulin boluses. As these patients did not need insulin injection reminders and yet accepted the intervention, they may have experienced these messages as way of extending the therapeutic interaction at home, between clinic visits.

Among previous studies based on message interventions for adolescents with type 1 diabetes, many are methodologically flawed [12, 13]. Few studies included a control group and used the HbA1c level as the primary outcome measure [18, 22]. The “Sweet Talk” study, reported in 2006, included 92 adolescents who were followed-up for 12 months. However, the SMSs were not the only component of the intervention [22]. In adults, two meta-analyses of studies demonstrated a significant effect of SMSs not only on glycemic control, but also on body mass index, diabetes self-care, and therapeutic patient education [33, 34].

Our study has two key strengths. The first is the use of SMSs as the sole means of intervention, enabling assessment of its impact versus standard of care. The second is the population made up exclusively of adolescents with poorly controlled type 1 diabetes which is not frequent [15, 23, 24], whereas for them the potential benefits could be great as they are particularly at risk of complications. Even a transient decline in HbA1c levels has been shown to be associated with a decreased risk of medium- and long-term complications of type 1 diabetes [35]; intensive insulin therapy has been reported to decrease the adjusted mean risks of retinopathy development by 76%, microalbuminuria by 39%, and clinical neuropathy by 60%; in another study, each 1% increase in HbA1c was associated with a 31% increase in the long-term risk of cardiovascular events, and the relative mortality rate increased exponentially for HbA1c values above 75 mmol/l (9%) [36].

Another novelty of our study is that our intervention is simple compared to those found in literature. Although the patients determined the message settings for number, time, and type of insulin at baseline, the messages were not otherwise personalized. We decided to focus on patients whose HbA1c level was above 80 mmol/l to constitute a homogeneous subgroup of patients as we know these patients experience great difficulties with treatment adherence and skip insulin injections. Noteworthy is our finding of a significant greater between-group difference in HbA1c levels in this subgroup with very poorly controlled type 1 diabetes in the intent-to-treat analysis. Thus, among those experiencing the greatest difficulties with treatment adherence, some patients may be particularly likely to respond to our simple method of SMS. Receiving SMSs may help the subset of non-adherent patients who want to follow their treatment but need continuous supported to change their behavior. In the same way, two recent studies reported that those who responded to text messages reminder had no deterioration in HbA1c [32, 37].

In the SMS arm, 20% of patients declined the intervention. This proportion is within the range considered acceptable when using the Zelen randomization method [26]). Of the remaining 36 patients, one third stopped the messages before the end of the trial. We decided to keep those patients who stopped the text messages in the SMS arm for the statistical analysis no matter the number and duration of the messages received, as the threshold at which a patient is to be considered as exposed to text messages is impossible to define. For those who refused or stopped our SMSs, better adherence to the SMS intervention could perhaps be obtained by tailoring the messages to their individual needs. A randomized trial in 366 old adolescents and adults with poor glycemic control (HbA1c>64 mmol/mol) showed that an individually tailored package of SMSs induced modest but statistically significant improvements in glycemic control [25].

One of the limitations of our study is that it is based on a single center, although our center is among the largest pediatric diabetology department in France. Secondly technical issues arose in 6 patients receiving messages, as reported by others [12]. Thirdly, despite the randomized design, we were unfortunate to have differences between the two groups regarding insulin pump use and median baseline HbA1c level. To mitigate for the HbA1c level difference, we did a post-hoc analysis and compared the two groups for the proportions of patients whose HbA1c level decreased by at least 1% between baseline and 6 months. Fourthly, some patients randomized to the intervention group declined to receive messages or discontinued the intervention. This non-compliant behavior is inherent to our study population since we are specifically addressing an adolescent population, furthermore a population with poor glycemic control. Fifth, the wide participant age range leads to a diverse study population, although very few patients were aged between 19 and 21 years of age. Sixth, concomitant use of any other diabetes apps during this study was not enquired, but this should have little impact on the outcome as patients were randomized.

Strengths of our study include the randomized design with a control group of patients who were not aware that an intervention was being used in other patients. The SMSs were the only intervention, so that all differences between the groups can be ascribed to them. Our study is an efficiency study and not a pilot study of feasibility [14, 20, 38]. The primary outcome measure was objective and reliable [1]. The sample size and follow-up duration place our trial among the most informative.

## Conclusions

While our pragmatic randomized study showed a positive trend among patients receiving text messages, no firm conclusions may yet be drawn regarding glycemic control, due to above mentioned limitations. Yet text messages seem to have a positive impact elsewhere on these particularly challenging adolescents as a significant proportion of them wanted to continue to receive messages, which could translate their desire of extending the therapeutic interaction at home, between clinic visits. Thus we believe that personalized messages can be a relevant additional tool to support behavior change among poorly controlled type 1 diabetic adolescents, in addition to the comprehensive care they receive.

## Supporting information

S1 Checklist CONSORT 2010 checklist of information to include when reporting a randomised trial*. (DOC)

S1 File (DOCX)
