# Supplementary material for: Text message reminders for adolescents with poorly controlled type 1 diabetes: A randomized controlled trial
Source: PLoS One. 2021 Mar 15;16(3):e0248549. doi: 10.1371/journal.pone.0248549 (PMC7959392; doi:10.1371/journal.pone.0248549)
Supplement: S1 File — (DOCX) [file pone.0248549.s002.docx]

Impact of Mobile Phone Short Text Messages on the Glycaemic Control of Adolescents with Poorly Controlled Type 1 Diabetes.

RESEARCH

PROTOCOL AIMING TO EVALUATE STANDARD CARE

Version no. 3.0 dated 18/06/2015

Biomedical Research ID (ID-RCB): 2014-A00915-42

Coordinating Investigators: Professor Michel Polak and Dr Jacques Beltrand

Paediatric Endocrinology Department

CHU de Necker

149 rue de Sèvres

75015 Paris

Scientific Director: Professor Jean Marc Treluyer and Dr Nour Ibrahim

Clinical Investigation Centre and Clinical Research Unit

CHU de Necker

149 rue de Sèvres

75015 Paris

Methodologist: Nelly Briand

Clinical Research Unit

Hôpital Necker-Enfants Malades

149, rue de Sèvres

75015 PARIS

#

Scientific Advisory Board

- Coordinating Investigator: Professor Michel Polak
- Scientific Director: Professor Jean-Marc Treluyer, Nour Ibrahim
- Statistical Analysis: Nelly Briand

Standard care research protocol

signature page

Research code: 2014-A00915-42

Title: “Impact of Mobile Phone Short Text Messages on the Glycaemic Control of Adolescents With Poorly Controlled Type 1 Diabetes.”

Version no. 3.0 dated 18/06/2015

The study will be conducted in accordance with the protocol, and current legislative and regulatory provisions.

| Coordinating investigator: |  |
| --- | --- |
| Professor Michel Polak  Paediatric Endocrinology Department  Hôpital Necker  149 rue de Sèvres  75015 Paris | Date: ……………/………/………..  Signature: |
| Scientific Director: |  |
| Professor Jean Marc Treluyer  Paris Centre Clinical Research Unit  Hôpital Necker  149 rue de Sèvres  75015 Paris | Date: ……………/………/………..  Signature: |
|  |  |
|  |  |

*The study received a favourable opinion from the Ile de France II CPP (IRB/IEC) on 17/11/2014*

Table of contents

[**SYNOPSIS** 7](#_Toc27144558)

[**GENERAL STUDY FLOW CHART** 10](#_Toc27144559)

[1. INTRODUCTION AND STUDY RATIONALE 11](#_Toc27144560)

[1.1. LITERATURE DATA 11](#_Toc27144561)

[1.1.1. Epidemiology. 11](#_Toc27144562)

[1.1.2. Diabetes and adolescence 11](#_Toc27144563)

[1.1.3. Insulin treatment: injections or pumps? 12](#_Toc27144564)

[1.1.4. Long-term complications. 12](#_Toc27144565)

[1.1.5. Current state of knowledge on text messaging intervention 13](#_Toc27144566)

[1.2. EXPECTED RESULTS 15](#_Toc27144567)

[1.3. DESCRIPTION OF THE STUDY 15](#_Toc27144568)

[1.3.1. Evidence that the medical strategies associated with the study, procedures performed and methods used comply with standard practice 15](#_Toc27144569)

[1.3.2. Justification that the specific monitoring procedures introduced by the study only entail negligible risks and constraints 16](#_Toc27144570)

[2. STUDY OBJECTIVE 16](#_Toc27144571)

[2.1. HYPOTHESIS TESTED 16](#_Toc27144572)

[2.2. PRIMARY OBJECTIVE 16](#_Toc27144573)

[2.3. SECONDARY OBJECTIVES 16](#_Toc27144574)

[3. MEDICAL STRATEGIES AND PROCEDURES CORRESPONDING TO STANDARD PRACTICE ASSESSED BY THE STUDY 17](#_Toc27144575)

[4. STUDY DESIGN 17](#_Toc27144576)

[4.1. TYPE OF STUDY 17](#_Toc27144577)

[4.2. STUDY DURATION 17](#_Toc27144578)

[4.3. RANDOMISATION ARM ALLOCATION 18](#_Toc27144579)

[4.4. EXPERIMENTAL DESIGN 18](#_Toc27144580)

[4.4.1. Randomised trial 18](#_Toc27144581)

[4.4.2. Rationale for the modified Zelen design: 18](#_Toc27144582)

[4.4.3. Procedures for limiting missing data 19](#_Toc27144583)

[4.5. ENDPOINTS 19](#_Toc27144584)

[4.5.1. Primary endpoint 19](#_Toc27144585)

[4.5.2. Secondary endpoints 19](#_Toc27144586)

[5. STUDY IMPLEMENTATION 19](#_Toc27144587)

[5.1. ASSESSMENT SCHEDULE FOR THE DIFFERENT GROUPS 19](#_Toc27144588)

[5.2. VISIT V1 - INCLUSION VISIT 19](#_Toc27144589)

[5.3. FOLLOW-UP VISITS at 3 and 6 months (M3 and M6) 20](#_Toc27144590)

[Summary table of the study time-line 21](#_Toc27144591)

[5.4. WITHDRAWAL FROM THE TRIAL AND PREMATURE WITHDRAWAL 21](#_Toc27144592)

[5.5. DISCONTINUATION OF THE STUDY 22](#_Toc27144593)

[6. STUDY POPULATION 22](#_Toc27144594)

[6.1. INCLUSION CRITERIA 22](#_Toc27144595)

[6.2. EXCLUSION CRITERIA 22](#_Toc27144596)

[6.3. STUDY WITHDRAWAL CRITERIA 22](#_Toc27144597)

[7. PATIENT INFORMATION PROCEDURES 23](#_Toc27144598)

[7.1. Patient information 23](#_Toc27144599)

[7.2. (Non)-objection by the patient or his/her representative to participation in the study 23](#_Toc27144600)

[8. MANAGEMENT OF ADVERSE EVENTS 23](#_Toc27144601)

[8.1. Adverse events (i.e. complications) related to the procedures, or combination of preventive, diagnostic or therapeutic strategies and procedures within the scope of standard treatment practice (other than medication). 23](#_Toc27144602)

[8.2. Adverse events related to the specific monitoring procedures introduced by the study 24](#_Toc27144603)

[9. DATA MANAGEMENT 24](#_Toc27144604)

[9.1. Right of access to data and source documents 24](#_Toc27144605)

[9.1.1. Access to data and confidentiality 24](#_Toc27144606)

[9.1.2. Source data and documents 24](#_Toc27144607)

[10. QUALITY CONTROL AND QUALITY ASSURANCE 25](#_Toc27144608)

[10.1. Instructions relating to data collection 25](#_Toc27144609)

[10.2. Quality control 25](#_Toc27144610)

[10.3. Audit 25](#_Toc27144611)

[11. DATA PROCESSING AND ARCHIVING OF STUDY DATA AND DOCUMENTS 25](#_Toc27144612)

[11.1. Data processing and archiving of study data and documents 25](#_Toc27144613)

[11.2. Archiving of study data and documents 26](#_Toc27144614)

[12. STATISTICAL CONSIDERATIONS 26](#_Toc27144615)

[12.1. Rationale for the sample size 26](#_Toc27144616)

[12.2. Description of planned statistical methods, including the planned interim analysis schedule 26](#_Toc27144617)

[13. ETHICAL AND LEGAL ASPECTS 27](#_Toc27144618)

[13.1. Declaration indicating that the study will be conducted in compliance with the protocol and current legislative and regulatory provisions 27](#_Toc27144619)

[13.2. Ethical evaluation of the specific monitoring procedures stipulated by the protocol 27](#_Toc27144620)

[14. LEGAL OBLIGATIONS 27](#_Toc27144621)

[14.1. Role of the administrator 27](#_Toc27144622)

[14.2. Submission to the CPP (IRB/IEC) 28](#_Toc27144623)

[14.3. Authorisation by the French Data Protection Authority (CNIL) 28](#_Toc27144624)

[14.4. Substantial amendment to the protocol 28](#_Toc27144625)

[14.5. Final study report or publication 28](#_Toc27144626)

[14.6. Rules relating to publication 28](#_Toc27144627)

[15. LIST OF APPENDICES 29](#_Toc27144628)

# **SYNOPSIS**

| Title | Impact of Mobile Phone Short Text Messages on the Glycaemic Control of Adolescents With Poorly Controlled Type 1 Diabetes. |
| --- | --- |
| Coordinating Investigator | Professor Michel Polak |
| Investigating Centre | Paediatric Endocrinology Department, Hôpital Necker |
| Type of standard care assessed | Text message sent to adolescents reminding them to administer their insulin injection |
| Study duration | 1 year |
| Patient follow-up | 6 months |
| Target population | Adolescents aged between 12 and 20 years inclusive, with inadequately controlled type 1 diabetes |
| Study objective | The primary objective of this study is to assess whether intervention by sending a text message to adolescents with inadequately controlled type 1 diabetes reduces the HbA1c level by 1% at 6 months.  Secondary objectives:   - Measurement of compliance at 3 and 6 months. - Measurement of quality of life at 3 and 6 months - Changes in HbA1c level at 3 months |
| Methodology | Prospective randomised single-centre interventional standard care study. Randomisation as per the Zelen method. |
| Planned number of centres | 1 |
| Planned number of patients | 100 subjects will be included  50 patients in group G1 = Standard management  50 patients in group G2 = Text messaging |
| Selection criteria | Inclusion criteria:   - aged between 12 and 20 years inclusive - type 1 diabetes - diagnosed more than 6 months previously - HbA1c level at enrolment greater than or equal to 8.5% - for minors: no objection by a person with parental authority - for adults: no objection by the patient - previous medical examination (Art. L.1121-11 of the French Public Health Code) - able to be followed up for 6 months following inclusion. - patient registered with or covered by a health insurance scheme - adolescents with a mobile phone able to receive text messages (SMS)   Exclusion criteria:   - aged under 12 years or over 21 years - with a HbA1C level at enrolment < 8.5%. - pregnancy - serious psychiatric comorbidity, such as serious personality disorder or psychosis. - patient and/or parents unable to understand or speak French, hence unable to understand the non-objection form. |
| Criteria for premature withdrawal | - withdrawal of consent - decision by the patient or parents to no longer be followed up in the study |
| Study implementation | Adolescents with type 1 diabetes whose current glycated haemoglobin level is greater than or equal to 8.5% are included and randomised to two groups, then followed up for 6 months:   - patients with standard follow-up (G1), - patients with standard follow-up and receiving text messages (SMS) (G2).   V0- Inclusion visit   - Verification of eligibility criteria - Invitation to take part in the study. - Collection of the non-objection form for participation in a cohort on compliance (IL1) - Randomisation. - Only adolescents randomised to group G2 are informed that they will receive text messages (SMS) (Zelen method). - Collection of the non-objection form for participation in the text messaging (SMS) study if the patient is randomised to the investigational arm (IL2) - Inclusion - Clinical examination - Blood sample: assay of HbA1c level - Completion of quality of life questionnaire - Completion of inclusion form   M3 – 3-month visit   - Clinical examination - Blood samples for laboratory work-up (assay of HbA1c level) - Completion of M3 follow-up form - Completion of quality of life questionnaire - Collection of blood glucose monitoring diary and distribution of a second diary   M6 – 6-month visit:   - Clinical examination - Blood samples for laboratory work-up (assay of HbA1c level) - Completion of M6 follow-up form. - Collection of capillary blood glucose diary distributed at M3. - Completion of quality of life questionnaire - Satisfaction survey for patients randomised to the text messaging arm   Text messaging characteristics:   - A text message will be sent every day. - At the usual insulin injection times based on the patient’s personal therapeutic regimen. - Example of message content:   *“Remember to administer your slow-acting insulin injection.”*  *“You are about to have your meal; remember to administer your rapid-acting insulin injection (or bolus - if a pump is used).”*  *“Remember to administer your mixed insulin injection.”* |
| Primary and secondary endpoints | Primary endpoint   - HbA1c level at 6 months   Secondary endpoints   - HbA1c level at 3 months - Measurement of compliance at M3 and M6: blood glucose monitoring diary - Inclusion, M3 and M6 quality of life questionnaire. - Satisfaction survey for patients randomised to the text messaging arm |
| Statistical analysis | Analysis of the primary endpoint: comparison of HbA1c level at 6 months between patients having received text messages and patients not having received reminder text messages.  Analysis of the secondary endpoints: comparison of HbA1c level at 3 months, compliance and quality of life between patients having received text messages and patients not having received reminder text messages, analysis of the satisfaction survey. |

# **GENERAL STUDY FLOW CHART**

Inclusion visit

Non-objection to participation in a 6-month cohort study

Randomisation

Control arm G1:

Standard management

Investigational arm G2:

Daily text messaging

Non-objection

to receiving text messages

Objection

Non-objection

Standard management

Text messaging

Assessment at 3 months and 6 months

|  | V0 | M3 | M6 |
| --- | --- | --- | --- |
| Verification of eligibility | X |  |  |
| Information | X |  |  |
| Non-objection to participation in a cohort study | X |  |  |
| Randomisation | X |  |  |
| Non-objection to receiving texts for adolescents in group G2 | X |  |  |
| Clinical examination | X | X | X |
| Clinical form to be completed | X | X | X |
| Laboratory work-up (HbA1c) | X | X | X |
| Distribution of blood glucose monitoring diary | X | X |  |
| Collection of blood glucose monitoring diary |  | X | X |
| Quality of life questionnaire | X | X | X |
| Satisfaction survey (only for patients in the text messaging arm) |  |  | X |

# INTRODUCTION AND STUDY RATIONALE

## LITERATURE DATA

### Epidemiology.

The following data originate from the “Entred-Enfant” and “Entred-Ado” surveys conducted between 2007 and 2010, and were published by the French National Institute for Public Health Surveillance (InVS).

The incidence of type 1 diabetes in France shows a constant upward trend, increasing from 8 per 100,000 children aged under 15 in 1988 to 15 per 100,000 in 2007. The annual increase is estimated at 3.3%. Higher growth is observed in the youngest children (7.6% among 0-4 year-olds). Hence, mean age at diagnosis is continuing to fall.

In 2010, the number of children with type 1 diabetes in France was estimated at approximately 12,000. The mean age of these diabetic patients corresponded to 12 years, with 68% aged between 11 and 17 years (i.e. approximately 8,200 adolescents). Most of these adolescents show satisfactory blood glucose control with a mean HbA1C level of 8%.

No studies estimating the number of adolescents with inadequately controlled type 1 diabetes currently exist.

Lastly, again, according to the same survey, 90% of adolescents are followed up in a hospital setting.

### Diabetes and adolescence

Achieving satisfactory control of diabetes in adolescents faces numerous major challenges. The serious complications of type 1 diabetes are closely related to metabolic balance in the first 10-20 years of the disease, particularly in adolescence. Furthermore, the Entred-Ado survey showed that diabetes has an impact on schooling in this patient population, with a higher proportion of pupils being kept down a year compared to their peers.

Although effective treatments for type 1 diabetes are available, treatment adherence among adolescents is lower than for other paediatric age groups. Hence, adolescents face a number of obstacles with regard to compliance.

Joshua S. Borus and Lori Laffel [29] describe three main obstacles to compliance in this population. Firstly, adolescence is a period in which young people are preoccupied with belonging to a peer group. Patients worry that their condition will make them different and cause them to be viewed negatively by their peers. At the same time, inadequate compliance can be part of the natural tendency at this age to take risks. Furthermore, up to 15% of adolescents with type 1 diabetes have suffered from depression at some point in their lives. A study among diabetic children conducted by A. Andronikof-Sanglade, based on the Rorschach test, mentions a fundamental narcissistic flaw. Treatment of diabetes is restrictive and can be perceived as inconvenient or intrusive, repeatedly interrupting the empowerment process which had been progressing naturally up to that point. Lastly, this population is at higher risk of developing eating disorders which have a negative impact on compliance.

### Insulin treatment: injections or pumps?

Two treatment procedures currently exist: syringes and insulin pumps.

Syringes and injector “pens” allow insulin to be delivered several times a day. The most widely used forms of insulin in a paediatric setting are rapid-acting insulin analogues, intermediate-acting insulin and slow-acting insulin analogues.

The most widespread regimens are as follows [17]:

- treatment regimen with two injections: mixture of rapid-acting insulin and intermediate-acting insulin prepared immediately before use in an insulin syringe, before breakfast and the evening meal.

- treatment regimen with three injections: an injection before an afternoon snack or before lunch is added to the treatment regimen comprising two injections.

- basal-bolus treatment regimen: a rapid-acting analogue injection before each meal and a slow-acting insulin analogue injection before bedtime.

Increasingly used in a paediatric setting, insulin pumps are portable devices which administer continuous insulin infusions. The insulin doses are adapted according to capillary blood glucose levels and glycated haemoglobin measured every 3 months.

The insulin pump replaces the multiple treatment injections using insulin “pens”, and thus has numerous advantages. However, monitoring of capillary blood glucose is very important as even minor pump failure can lead to a risk of ketoacidosis. In the event of pump failure, the body has no insulin stores and ketone bodies rapidly increase. The patient should react promptly in the event of unexplained hyperglycaemia. Hence, the patient should receive thorough therapeutic education beforehand and comply with instructions.

1.1.3 Acute complications.

Two acute complications are a cause for concern. Firstly, hypoglycaemia when insulin intake is excessive relative to the ingested carbohydrates. Symptoms typically include malaise, visual disturbances, sudden fatigue and abdominal pain.

Secondly, ketoacidosis is the complication feared in adolescents. This occurs when insulin intake is insufficient. In this situation, the cells are deficient in glucose, the main source of energy. The body then uses fatty acids to produce energy. In return, ketone bodies accumulate, leading to ketoacidosis.

### Long-term complications.

The serious complications of type 1 diabetes are closely linked to factors including metabolic balance during adolescence. Clinical practitioners, moreover, experience difficulties due to limited understanding, among some adolescents, of the significance of long-term complications.

Patients with type 1 diabetes can be affected by four types of long-term complications: microangiopathy, autoimmune disease, cardiovascular complications and psychiatric comorbidities.

Microangiopathy may affect the kidneys, retina or nervous system. The first symptom of nephropathy is the onset of microalbuminuria. Retinopathy usually develops after 5-10 years of disease progression. Diabetic neuropathy is rare in the paediatric population, but may nonetheless be observed in the event of long-standing and very poorly controlled disease.

The types of autoimmune disease most frequently associated with type 1 diabetes are as follows in order of frequency: thyroid dysfunction, coeliac disease and Addison’s disease.

Paediatricians should pay greater attention to blood pressure (HBP) in these patients. BP ≥ 95th percentile for age, gender and height, repeatedly over 3 consecutive days indicates HBP. Dyslipidaemia should be investigated in the event of a family history of dyslipidaemia or cardiovascular disease.

Patients should undergo regular foot examinations from adolescence, which is an opportunity to educate patients in good local hygiene.

Lastly, attention should be given to psychiatric comorbidities, more common in these patients, and which warrant routine psychological management: depression, suicide attempt, eating disorders and high-risk behaviour.

### Current state of knowledge on text messaging intervention

A/ Adults

To our knowledge, no approved therapeutic education programmes based on text messaging currently exist for adults with type 1 diabetes. Nevertheless, numerous studies on this type of intervention have been conducted alongside adults with chronic disease.

In September 2012 [24], a Dutch team published a randomised study conducted in 104 patients with type 2 diabetes, receiving oral antidiabetic agents and undergoing real-time electronic monitoring. Two groups were created: a group receiving a reminder text message in case they missed their treatment, and a group not receiving text messages. At 6 months, patients receiving text messages showed significantly greater compliance, and the patients found this type of intervention to be very acceptable.

In the context of chronic kidney disease, Chen SH. [25] conducted a randomised controlled trial in 54 patients over a total period of 12 months. Two groups were compared: text messaging support and a control group without text messaging support. Glomerular filtration rate was significantly higher in the text messaging group (29.11 +/- 20.61 *vs*. 15.72 +/- 10.67 mL/min; *p* < 0.05) and the number of hospital admissions was significantly lower in the same group.

B/ Adolescents

As text messaging is one of the preferred communication methods among adolescents, several teams tested this approach as a new way of reaching this population. This approach has the advantage of being reasonably priced and allowing for large-scale implementation.

1. Disorders other than type 1 diabetes

The text messaging intervention evidenced superior results for the prevention of excessive alcohol use in adolescents. A Swiss study published in 2013 [26] focused on the impact of individual intervention via text messaging (1-3 texts per week, for 3 months), and via a website, on alcohol use among school-age adolescents. The text message content depended on their level of alcohol use. In total, 364 adolescents took part in the study. This approach significantly reduced the number of high-risk episodic use and the number of units of alcohol consumed per week. The authors also noted that young people found this approach highly acceptable

Numerous teams have evaluated the efficacy of text messaging as an additional therapeutic measure in the management of obese adolescents. This type of intervention has not demonstrated long-term efficacy as shown by the US study “The Loozit Study” published in March 2013 [27]. This randomised control trial conducted in 151 overweight adolescents aged 13-16 years showed that the addition of supplementary therapeutic support via telephone calls/text messages/email had no effect at 24 months.

Lastly, in 2010 [28] Gentles JC published a literature review aiming to evaluate the impact of new technologies (websites, text messages, intranet, video conferences, emails) on communication between families of paediatric patients suffering from chronic disorders and healthcare teams. The disorders concerned were asthma, type 1 diabetes, psychiatric disorders and multiple disabilities. Patient age was between 2 and 12 years. The results were controversial. The authors highlighted the need for more practical interventions from a methodological perspective.

2. Type 1 diabetes

Text messaging could be a way to overcome obstacles in terms of compliance among diabetic adolescents. Hence, numerous teams wished to evaluate the efficacy of this approach in this indication.

Again in the United States, Mulvaney [5] conducted a study among adolescents with type 1 diabetes. They received approximately 10 text messages per week. The content aimed to motivate the patients and remind them to take their treatment over a 3-month period. This intervention significantly reduced the HbA1c level (9.9% in the control arm *vs*. 8.8% in the text messaging arm). However, the sample size was small (n=23).

In the United Kingdom, in 2013 [4] Louch showed that daily personalised text messaging support targeting young adults with diabetes (n=18) significantly improved treatment compliance (increase in the number of patient-reported insulin injections administered).

In the United States, Hanauer DA. initiated a scheme known as CARDS (computerized automated reminder diabetes system) in 2009. Patients were randomised to two groups: they received an automated reminder to monitor their capillary blood glucose, either by text message (n=22) or by email (n=18). Patients could then enter their capillary blood glucose results and schedule the next reminders. Patients randomised to the text messaging arm recorded more capillary blood glucose results and scheduled more reminders at 1 month. This difference became less marked at 3 months. This population nonetheless found this intervention acceptable.

Joshua S. Borus and Lori Laffel [29] suggest that interventions requiring additional contribution by adolescents can prove to be ineffective due to having an even greater impact on adolescents’ daily lives.

In Scotland, “The Sweet Talk Trial” was conducted in 2010 over a 12-month period, and randomised 64 adolescents and young adults to three groups: standard treatment, standard treatment with “Sweet Talk”, together with intensive insulin therapy and “Sweet Talk”. Patients randomised to the “Sweet Talk” arm also received individual daily text messages as well as text messages referring to their general therapeutic regimen. Although text messaging did not in itself cause a reduction in HbA1c levels, it is nonetheless associated with an increase in self-efficacy and superior compliance reported by patients.

Benhamou published “The Pumpnet Study” [8] in 2007, in France. 15 patients with insulin pumps received weekly medical advice by text messaging over a 12-month period. The author observed an improvement in quality of life (DQOL) and a non-significant reduction in HbA1c level (-0.25+/-0.94%, *p* <0.10) at 12 months.

Lastly, a study conducted in Saudi Arabia, published in March 2014 [12], showed promising results. It was conducted over a 6-month period in 200 children and adolescents with type 1 diabetes. The patients received different types of text messaging support (information messages, interactive messages and video messages). The authors evidenced a significant reduction in fasting blood glucose (from 150 mg/L to 133 mg/L, *p* < 0.001), post-prandial blood glucose, HbA1c level and onset of hypoglycaemia in these patients in comparison with the same parameters prior to the study. This study did not, however, have a control group.

In view of these controversial results, we therefore wish to conduct a study in adolescents with inadequately controlled type 1 diabetes, with a view to testing the hypothesis according to which support via individual text message reminders prior to each insulin injection over a 6-month period may significantly improve metabolic control of diabetes.

## EXPECTED RESULTS

This project aims to offer a practical and simple solution for doctors treating adolescents with inadequately controlled type 1 diabetes.

## DESCRIPTION OF THE STUDY

This protocol falls within the scope of a study aiming to assess standard care, as defined by French law no. 2004-806 of 9 August 2004 on public health policy, and its implementing decree (no. 2006-477) of 26 April 2006. (Reference texts: Articles L.1121-1, paragraph 2, and R1121-3 of the French Public Health Code).

### Evidence that the medical strategies associated with the study, procedures performed and methods used comply with standard practice

The number of mobile phone lines in France is currently equal to the number of inhabitants. According to [the latest figures from the telecommunications regulatory authorities, at the end of December 2010,](http://www.arcep.fr/index.php?id=10743#c17814) 64.4 million SIM cards were registered in France, i.e. a 4.9% increase in one year.

### Justification that the specific monitoring procedures introduced by the study only entail negligible risks and constraints

The additional procedures introduced by the study correspond to automated standard text messages sent to the patient and completion of a quality of life questionnaire.

| Monitoring procedures  within the treatment context  (Standard management) | Specific monitoring procedures  introduced by the study  (Additional procedures compared with standard management) |
| --- | --- |
| Guidance for compliance every 3 months. | Daily text messaging over a 6-month period  Completion of a quality of life questionnaire  Completion of a satisfaction survey |

Table 1: Specific monitoring procedures within the treatment context/introduced by the study

These procedures do not entail any risks, and the associated constraints are negligible. The self-administered questionnaire should take approximately five minutes to complete.

# STUDY OBJECTIVE

## HYPOTHESIS TESTED

Daily text messaging support for adolescents with inadequately controlled type 1 diabetes may improve treatment compliance and therefore blood glucose control.

## PRIMARY OBJECTIVE

The primary objective of this study is to assess whether intervention by sending a text message to adolescents with inadequately controlled type 1 diabetes, over a 6-month period, significantly reduces the HbA1c level.

## SECONDARY OBJECTIVES

The secondary objectives fall into four categories.

Firstly, we will assess HbA1c level after 3 months of follow-up.

Secondly, we will measure compliance via the blood glucose diary at 3 months and 6 months after the start of the study. This diary is given to the patient at the start of the study, then at the 3-month study visit. Thirdly, we would like to assess quality of life among adolescents based on a questionnaire given to the patients at inclusion, at 3 months and 6 months after the start of the study (PedsQL questionnaire version 4.0). Lastly, we would like to conduct a satisfaction survey alongside adolescents randomised to the text messaging arm at 6 months of participation in the study.

# MEDICAL STRATEGIES AND PROCEDURES CORRESPONDING TO STANDARD PRACTICE ASSESSED BY THE STUDY

The strategy assessed will correspond to automated text messaging.

- Number of text messages:

Patients will receive several text messages daily, reminding them to administer their insulin injection; no text messages will relate to capillary blood glucose levels.

- Text messaging times:

Text messages will be sent at the usual insulin injection times based on the patient’s personal therapeutic regimen. Hence, the clinical practitioner including the patient in the study should note the times at which the text messages should be sent (refer to APPENDIX 6).

- Text message content:

A single text message can be no longer than 160 words; hence, the messages sent will be as follows, for example:

- Message content concerning slow-acting insulin analogue injections: “Remember to administer your slow-acting insulin injection.”

- For rapid-acting insulin analogues or bolus regimens when a pump is used: “You are about to have your meal; remember to administer your rapid-acting insulin injection.”

- For mixtures of rapid-acting insulin and intermediate-acting insulin prepared immediately before use: “Remember to administer your mixed insulin injection.”

The telephone contact details, inclusion number (but not the full identity) of all patients randomised to the “text messaging” arm, together with the date and time of text messaging, will be forwarded to a call centre to schedule automated text messaging. Patients in the control arm will not receive text messages. All patients in the investigational (text messaging) arm and control (no text messaging) arm will otherwise receive the same care:

- Information on the study
- Clinical examination
- Quarterly monitoring of HbA1c level
- Clinical form
- Usual doctor’s advice
- Distribution and collection of the blood glucose diary
- Completion of a quality of life questionnaire at 3 months and at the end of the study.

# STUDY DESIGN

## TYPE OF STUDY

This is a prospective, randomised, open-label, single-centre standard care study. Randomisation will be carried out according to the modified Zelen method.

## STUDY DURATION

The total study duration is 18 months. The inclusion period is 12 months. The length of participation in the study for each patient will be 6 months.

## RANDOMISATION ARM ALLOCATION

The randomisation list will be drawn up using blocks of varying sizes. The list and size of the blocks will not be communicated to the investigators. Concealed allocation will take place using a computerised randomisation system.

## EXPERIMENTAL DESIGN

### Randomised trial

Two patient groups will be studied: the investigational group which will receive text messages (G2) and the control group which will receive standard management (G1).

The randomised controlled trial is the reference method for assessing treatment. Randomisation is the only method which yields comparable groups for known and unknown prognostic factors and limits selection bias.

The comparator will correspond to the patients’ standard management by their diabetes specialist.

### Rationale for the modified Zelen design:

The primary endpoint is HbA1c level at 6 months. Changes in this level are related to patient compliance. One of the secondary endpoints (satisfaction, quality of life) is a patient-reported endpoint which, by definition, is totally subjective. A recent study showed that, in the absence of blinding, the treatment effect is overestimated for randomised trials with a subjective endpoint (Wood L, BMJ 2008). In order to limit bias related to the absence of blinding, we propose to use a modified Zelen design.

We will use the Zelen method while obtaining agreement to take part in two stages. All eligible patients and their parents will initially be invited to take part in a cohort study aiming to assess changes in diabetes control over a 6-month period. An information leaflet (IL1) on the study objectives and procedures will be given to the parents and young patients. Patients will be informed that they may choose not to take part in the study. Secondly, still during the first appointment, after the randomisation step, adolescents randomised to the interventional group, and their parents, will be invited to take part in a study assessing text messaging intervention. A second information leaflet (IL2) on the study objectives and text messaging intervention procedures will be given to the parents and young patients. Patients will be informed that they may choose not to take part in this study.

This method makes it possible to avoid the risks of bias due to the absence of blinding. In a conventional randomised trial, patients assigned to the control arm may modify their behaviour as they know they will not receive text messaging support.

This design is acceptable in this context for several reasons: The procedure for obtaining consent in two stages has already been used to assess rehabilitation, and several ethics committees have approved its use in this context (Quilty *et al*., 2003, Rannou, PHRC National in press, Forestier R, ARD 2009).

From a methodological perspective, the limitations of the Zelen design correspond to contamination risks due to refusal of intervention. In our study, the contamination risk should not be high in view of:

- The absence of risk related to intervention

- The short intervention time-frame

- Contamination risk in the standard management group is very unlikely as this text messaging intervention is not currently proposed outside the scope of this study.

### Procedures for limiting missing data

No or very few patients will be “lost to follow-up”, in view of their age and their usual obligations in terms of medical follow-up. The number of missing data should be very low.

## ENDPOINTS

### Primary endpoint

HbA1c, quantifying blood glucose control, will be used to define the primary endpoint. An improvement in blood glucose control is defined as a 1% reduction in HbA1c at 6 months.

### Secondary endpoints

The secondary endpoints are as follows:

- Improvement in blood glucose control at 3 months
- Measurement of compliance: by assessing the capillary blood glucose diary, at 3 months and at the end of the study.
- Measurement of quality of life among the adolescents: anonymous self-administered questionnaire distributed, at inclusion, at 3 months and at the end of the study (PedsQL questionnaire version 4.0).
- Satisfaction survey after 6 months of participation in the study.

# STUDY IMPLEMENTATION

An inclusion visit and two follow-up visits are planned. Patient inclusion and follow-up will take place at the patients’ usual treatment centres.

## ASSESSMENT SCHEDULE FOR THE DIFFERENT GROUPS

The principal endpoint will be assessed at the inclusion visit and during the follow-up visits at 3 and 6 months. The secondary endpoints will be assessed at the follow-up visits at 3 and 6 months.

## VISIT V1 - INCLUSION VISIT

The investigator will inform parents of potentially eligible minor patients and will invite their child to take part in the cohort study aiming to assess changes in diabetes control over a 6-month period (Information leaflet 1_ APPENDICES 8 and 9). Adult patients will be informed by the investigator directly.

Non-objection by the parents and/or adolescents will be documented in the patient’s medical record (date on which the information was provided, objection expressed or not, and signature of the person in charge of the visit).

Clinical examination

After verifying the eligibility criteria and non-objection to participation in the study, the investigator will collect the medical history and clinical information usually taken into account in the management of diabetic patients:

- medical history
- history of psychiatric disorders
- date of diabetes diagnosis
- current treatment regimen and insulin analogue injection times

Laboratory tests

Glycated haemoglobin (HbA1c) levels will be measured according to the method used at Centre Hospitalier de Necker.

Randomisation

- Patients randomised to the “text messaging” investigational arm: a second information leaflet (IL2- APPENDICES 10 and 11) on the study objectives and text messaging intervention procedures will be given to the parents and/or young patients. If patients refuse the text messaging intervention, the patients will receive conventional follow-up. Non-objection by the parents and/or adolescents to the text messaging intervention will also be documented in the patient’s medical record (date on which the information was provided, objection expressed or not, and signature of the person in charge of the visit).
- Patients randomised to the “standard management” control arm will receive their usual care.
- Completion of the quality of life questionnaire for all patients

At the end of the visit, the investigator will send the information sheet for centralised data entry.

## FOLLOW-UP VISITS at 3 and 6 months (M3 and M6)

The following procedures will be performed during these visits:

- Clinical examination
- Blood samples for laboratory work-up (assay of HbA1c level)
- Collection of the capillary blood glucose diary
- Distribution of a second capillary blood glucose diary
- Completion of the quality of life questionnaire for all adolescents
- Collection of any adverse events
- Satisfaction survey for patients randomised to the text messaging arm at M6.

These visits will be held with the doctor who included the patient in the study, and at the usual treatment centre.

At the end of the visit, the investigator will send the information sheet for centralised data entry.

## Summary table of the study time-line

|  | Inclusion | M3 | M6 |
| --- | --- | --- | --- |
| Eligibility | X |  |  |
| Information | X |  |  |
| Non-objection | X |  |  |
| Randomisation | X |  |  |
| Clinical form to be completed | X | X | X |
| Clinical examination | X | X | X |
| Assay of HbA1c level | X | X | X |
| Distribution of blood glucose monitoring diary | X | X |  |
| Text message sent | X | X | X |
| Collection of blood glucose diary |  | X | X |
| Quality of life questionnaire | X | X | X |
| Satisfaction survey (text messaging arm) |  |  | X |

Table 2: Summary table of the study time-line

## WITHDRAWAL FROM THE TRIAL AND PREMATURE WITHDRAWAL

The participants will leave the study after the 6-month follow-up period.

The patient, or person with legal authority, may withdraw their participation in the study at any time, without their decision compromising their continued treatment, quality of care or relationship with their doctor in any way.

Any patients erroneously included and not fulfilling the inclusion and exclusion criteria will be prematurely withdrawn from the trial. Other deviations from the protocol will not be considered as study withdrawals.

The investigator should document the reasons for premature withdrawal as thoroughly as possible. Study withdrawals should notably be related to the following reasons:

- Refusal by the subject or his/her parents to continue taking part in the trial
- Patient included erroneously
- Patient lost to follow-up

If a participant does not attend the follow-up visits, the investigator responsible for the patient’s care should contact the patient to determine his/her condition at the time of withdrawal from the study. If it is impossible to contact the patient, s/he will be considered lost to follow-up at the date of the last visit.

Patients withdrawn from the trial cannot be re-included in the study. Their inclusion number will not be re-used.

## DISCONTINUATION OF THE STUDY

The decision may be made to discontinue the whole study prematurely due to:

- inadequate recruitment
- unresolved technical problems
- desire expressed by the investigators
- repeated and unjustified protocol violations

# STUDY POPULATION

In this study, patients aged 12-20 years are referred to as “adolescents”.

## INCLUSION CRITERIA

- aged 12-20 years
- child with type 1 diabetes
- type 1 diabetes diagnosed more than 6 months previously, HbA1c level > or equal to 8.5% at inclusion
- For minors: Non-objection by a parent and the patient
- For adults: Non-objection by the patient
- Children having had a previous medical examination (Art. L.1121-11 of the French Public Health Code)
- Able to be followed up for 6 months following inclusion.
- Patient registered with or covered by a health insurance scheme
- Adolescents with a mobile phone able to receive text messages (SMS)

## EXCLUSION CRITERIA

- Aged strictly below 12 years or 21 years or over
- Child with non-insulin-dependent diabetes.
- Pregnancy (declared)
- Serious psychiatric comorbidity, such as serious personality disorder or psychosis.
- Patient and/or parents unable to understand or speak French, hence unable to understand the non-objection form.

## STUDY WITHDRAWAL CRITERIA

The decision by the patient or a parent to no longer be followed up in the study represents a study withdrawal criterion.

# PATIENT INFORMATION PROCEDURES

In compliance with French law no. 2004-806 of 9 August 2004 on public health policy, the investigator is obliged to inform patients prior to their participation in a study aiming to assess standard care.

## Patient information

In compliance with Article R. 1121-3 of the French Public Health Code, persons who take part in a study are informed via a written document, submitted to the relevant ethics committee beforehand. When the patient is being included in the study, the patient or his/her representative will be given information on the study verbally or in writing, via the information leaflet distributed by the doctor.

When the study has ended, the person taking part in the study may be notified of the overall results of the study, in accordance with the procedures stated in the information leaflet.

## (Non)-objection by the patient or his/her representative to participation in the study

In the context of studies aiming to assess standard care, the patient has the option to object to participation in the study. This is stated in the information document stipulated in Article R1121-3 of the French Public Health Code. (refer to the French decree of 9 March 2007 laying down the contents of the application for an opinion from the Ethics Committee for studies aiming to assess standard care, mentioned in paragraph 2°, Article L. 1121-1 of the French Public Health Code).

Patient information and non-objection should be documented and dated in the medical record. If the patient objects to be included in the study, s/he will continue to receive standard management. Non-objection by only one of the two parents should be documented for a standard care protocol.

Non-objection by the parents and adolescents will be documented in the patient’s medical record: date on which the information was provided, objection expressed or not, and signature of the person in charge of the visit.

# MANAGEMENT OF ADVERSE EVENTS

## Adverse events (i.e. complications) related to the procedures, or combination of preventive, diagnostic or therapeutic strategies and procedures within the scope of standard treatment practice (other than medication).

In the context of studies aiming to assess standard care, the medical strategies or procedures, associated with the study, correspond to standard practice and are implemented in compliance with their indications. The potential adverse events are therefore incidents related to standard patient management and are not required to be specifically reported by the study administrator.

This study does not incur any additional risks, and no adverse events are expected in relation to text messaging.

No events will be reported in the context of this study.

## Adverse events related to the specific monitoring procedures introduced by the study

The specific monitoring procedures introduced by a study aiming to assess standard care only entail negligible risks and constraints for persons taking part in the study. Consequently, a procedure for managing adverse events is not required for this study. In this case, the endpoints will be recorded during a medical visit at 6 months. The study will not require any additional samples or imaging examinations. This study does not incur any additional risks, and no adverse events are expected No events will be reported in the context of this study.

# DATA MANAGEMENT

## Right of access to data and source documents

### Access to data and confidentiality

The administrator ensures that the protocol and information leaflet intended for patients included in the study or their representatives stipulate that the investigators shall allow access to the documents and personal data strictly required for monitoring, quality control and audits of a study aiming to assess standard care, by personnel individually appointed for this purpose by the study administrator. The administrator also ensures that each person taking part in the study has not objected to allowing access to their personal data.

These activities must comply with strict confidentiality regulations. Personnel thus appointed for these purposes are bound by professional secrecy, notably under the conditions defined in Articles 226-13 and 226-14 of the French Penal Code, in the same way as the investigators themselves.

During and at the end of the study, data collected on the participants and transferred to the administrator by the investigators (or any other study personnel) shall be coded. Under no circumstances shall these data clearly display the names or addresses of the persons concerned, or any other identifiable information. Data will be collected on each patient from the case report form. Patients will be allocated a study-specific code number, indicating the centre, order of inclusion and the patients’ initials (surname and first name).

Telephone contact details for all patients will be forwarded to a call centre. The study inclusion number, telephone number (without the patient’s name and address) together with the text messaging dates and times will be recorded on a secure web platform, separate from the case report form, allowing the text messages to be scheduled within the time-frames stipulated by the protocol. This platform will be accessed via a login and password. The information given to the service provider, namely the patient’s telephone contact details and inclusion number, will not be stored by the service provider but will be destroyed after the last text message has been sent.

The study database will only contain medical data (taken from the patient’s medical record at inclusion, at 3 months and at 6 months).

### Source data and documents

The source data are taken from the medical records of patients included in the study

# QUALITY CONTROL AND QUALITY ASSURANCE

## Instructions relating to data collection

All information required by the protocol should be entered in the case report forms. The data should be collected as and when they are obtained, and recorded in the case report forms in a legible manner. All missing data should be coded.

## Quality control

The investigator shall allow access to the documents and personal data strictly required for monitoring, quality control and audits of this study, by personnel responsible for quality control and duly appointed for this purpose by the administrator.

The person(s) appointed by the administrator regularly visit each centre, during study initiation, once or several times during the study according to the frequency of inclusions, and at the end of the study. The following aspects will be reviewed during these visits:

- patient protection and safety,
- compliance with the study protocol, the procedures defined therein and currently applicable regulatory texts,
- quality of the data collected in the case report form: accuracy, missing data, consistency of data with source documents (medical records, appointment diaries, original laboratory results, etc.),

All visits will be described in a written monitoring report.

## Audit

An audit may be carried out at any time by persons appointed by the [administrator and](http://www.chusa.jussieu.fr/urcest/sous_cadre.php?fich=lexique/new_index.php?isphp=0&fich=ec/legislation/dispositionslegislativespromoteur.htm) independent of the persons in charge of the study. It aims to guarantee the quality of the study, the validity of the results and compliance with the law and current regulations.

Personnel responsible for managing and overseeing the study agree to comply with the requirements stipulated by the administrator and the competent authorities with respect to audits or inspections concerning the study.

Audits may apply to all stages of the study, from development of the protocol to publication of the results and archiving of the data used or generated in the context of the study.

# DATA PROCESSING AND ARCHIVING OF STUDY DATA AND DOCUMENTS

## Data processing and archiving of study data and documents

Data entry is organised in a case report form, and the personnel in charge of data entry are the investigators and their teams. The data entered will be periodically checked by one of the investigators to ensure there are no missing data or inconsistencies.

The data will be checked by a data manager. Any “queries” will be issued. Prior to statistical analysis, the data manager will freeze the database. This database, used for the statistical analysis, will be archived by the person in charge of the analysis or the computer analyst (printed or electronic format).

## Archiving of study data and documents

“Source” documents will be kept in the patient’s medical record and archived under the responsibility of Hôpital Necker (AP-HP) (administrator). In keeping with the recommendations issued by the French Data Protection Authority (CNIL), the patient’s identity and contact details will not be communicated with his/her medical data. The patient will be identified by his/her initials (surname and first name), centre number and study inclusion number. The documents specific to a standard care study will be archived by the investigator until the findings are published.

This indexed archiving will include:

- Copies of the mandatory opinion from the CPP (IRB/IEC)
- The successive versions of the protocol (identified by the version no. and version date)
- All correspondence with the administrator
- The completed and validated case report form for each subject included
- All appendices specific to the study
- The final study report originating from the statistical analysis and quality control
- The certificates from any audits that may have been carried out during the study
- The database used for the statistical analysis must also be archived by the person in charge of the analysis (printed or electronic format).

# STATISTICAL CONSIDERATIONS

## Rationale for the sample size

Based on the results of the Hvidore study (Holl RW Eur J Pediatr 2003), we assume that the HbA1c level will follow a normal distribution, with a standard deviation of 1.6. The sample size necessary was calculated to evidence a difference in HbA1c level between the 2 “standard management” and “text messaging” groups of at least 1% after a 6-month follow-up period. Taking into account a type I error of 5% and 80% power, 41 subjects per group, i.e. 82 subjects in total, will need to be included and randomised.

To take into account a potential rate of refusal to take part in the “text messaging” arm, of approximately 10%, the sample size necessary was increased by 20%, i.e. 50 subjects per group, 100 subjects in total (Zelen, Stat med 1990).

Children who withdraw their consent, after being randomised to the “text messaging” arm will be followed up under the same conditions as those in the “standard management” group.

## Description of planned statistical methods, including the planned interim analysis schedule

The statistical analysis will be carried out using SAS software, under the supervision of Nelly Briand (Paris Centre CRU - Cochin-Necker). The statistical analysis will be carried out at the end of data collection, data entry and data consistency checks.

A descriptive analysis of the characteristics of the children included in each group will be performed first of all. Quantitative data are expressed as the mean ± standard deviation or median and range, and as the sample size and percentage for qualitative data.

The primary endpoint and secondary endpoints will be analysed on an intention-to-treat basis, i.e. all randomised patients will be analysed in their randomisation arm, irrespective of the intervention received. All tests used will be two-sided, with a 5% significance level.

*Main analysis*

The HbA1c level after the 6-month follow-up period will be compared between the “text messaging” and control groups using Student’s *t*-test.

*Secondary analyses*

The HbA1c level after the 3-month follow-up period will be compared between the “text messaging” and control groups using Student’s *t*-test.

For all secondary endpoints, satisfaction and compliance, two means will be compared using Student’s *t*-test or a Wilcoxon test if necessary. The percentages will be compared using the Pearson Chi-2 test or Fisher’s exact method, if necessary.

# ETHICAL AND LEGAL ASPECTS

## Declaration indicating that the study will be conducted in compliance with the protocol and current legislative and regulatory provisions

The administrator and person managing and overseeing the study undertake to ensure that the study is conducted in keeping with French law no. 2004-806 of 9 August 2004 on public health policy and currently regulatory provisions. (Articles L1121-1, paragraph 2° and R1121-3 of the French Public Health Code).

The data recorded during the study will be processed electronically in compliance with French law no. 78-17 of 6 January 1978 on data processing, data files and individual liberties, as amended.

The study will be conducted in compliance with the present protocol.

## Ethical evaluation of the specific monitoring procedures stipulated by the protocol

The specific monitoring procedures introduced by the study were subjected to ethical evaluation by the Ile de France II CPP (IRB/IEC). These procedures only entail negligible risks and constraints to persons taking part in this study.

# LEGAL OBLIGATIONS

## Role of the administrator

The administrator is defined by French law 2004-806 of 9 August 2004. Hôpital Necker (AP-HP) is the study administrator and the Paris Centre Clinical Research Unit carries out the regulatory duties associated with the study.

It submitted the application for an opinion to the relevant ethics committee.

## Submission to the CPP (IRB/IEC)

This study received a favourable opinion from the Ile de France II CPP (IRB/IEC) on 3 October 2014.

The opinion issued by the above-mentioned committee is stated in the information leaflet intended for the persons concerned.

## Authorisation by the French Data Protection Authority (CNIL)

This study is subject to French law no. 78-17 of 6 January 1978, as amended, relative to data processing, data files and civil liberties.

Only a normal declaration to the CNIL is required for single-centre studies.

Information on the rights of persons taking part in this study (right of access and rectification, and the right to object to the transfer of data covered by professional secrecy, liable to be used within the context of this study) is included int he patient information leaflet.

## Substantial amendment to the protocol

The study administrator shall submit any substantial amendments to the CPP (IRB/IEC) for opinion.

## Final study report or publication

The final study report or publication will be submitted to each investigator for opinion. The final version will be sent to the administrator as soon as possible after the effective study end date.

## Rules relating to publication

AP-HP retains ownership of the data, which cannot be used or transferred to a third party without its prior agreement.

The first signatories of the publications will be those persons who have effectively contributed to drawing up and implementing the protocol, and compiling the results.

AP-HP shall be mentioned in the connections of the author(s) of publications arising from this study, together with the AP-HP administrator

This study is registered at http://clinicaltrials.gov/ under number NCT02230137

# LIST OF APPENDICES

Appendix 1: References

Appendix 2: Investigators and associated teams

Appendix 3: Inclusion data collection form

Appendix 4: M3 data collection form

Appendix 5: M6 data collection form

Appendix 6: Blood glucose monitoring diary

Appendix 7: Control arm information leaflet - parents (IL1)

Appendix 8: Control arm information leaflet – adolescents and young adults (IL1b)

Appendix 9: Text messaging arm information leaflet - parents (IL2)

Appendix 10: Text messaging arm information leaflet - adolescents and young adults (IL2b)

Appendix 11: Satisfaction survey

Appendix 1: References

1. [Guljas R](http://www.ncbi.nlm.nih.gov/pubmed?term=guljas%20r%255bauthor%255d&cauthor=true&cauthor_uid=24269308), [Ahmed A](http://www.ncbi.nlm.nih.gov/pubmed?term=ahmed%20a%255bauthor%255d&cauthor=true&cauthor_uid=24269308), [Chang K](http://www.ncbi.nlm.nih.gov/pubmed?term=chang%20k%255bauthor%255d&cauthor=true&cauthor_uid=24269308), [Whitlock A](http://www.ncbi.nlm.nih.gov/pubmed?term=whitlock%20a%255bauthor%255d&cauthor=true&cauthor_uid=24269308). Impact of Telemedicine in Managing Type 1 Diabetes Among School-age Children and Adolescents: An Integrative Review. [J Pediatr Nurs.](http://www.ncbi.nlm.nih.gov/pubmed/24269308) 2013 Oct 31.
2. Franc S, Borot S, Ronsin O, Quesada JL, Dardari D, Fagour C, Renard E, Leguerrier AM, Vigeral C, Moreau F, Winiszewski P, Vambergue A, Mosnier-Pudar H, Kessler L, Reffet S, Guerci B, Millot L, Halimi S, Thivolet C, Benhamou PY, Penfornis A, Charpentier G, Hanaire H., [Telemedicine and type 1 diabetes: is technology per se sufficient to improve glycaemic control?](http://www.ncbi.nlm.nih.gov/pubmed/24139705)Diabetes Metab. 2014 Feb.
3. Herbert L, Owen V, Pascarella L, Streisand R., [Text message interventions for children and adolescents with type 1 diabetes: a systematic review.](http://www.ncbi.nlm.nih.gov/pubmed/23550554)Diabetes Technol Ther. 2013 May;15.
4. Louch G, Dalkin S, Bodansky J, Conner M., [An exploratory randomised controlled trial using short messaging service to facilitate insulin administration in young adults with type 1 diabetes.](http://www.ncbi.nlm.nih.gov/pubmed/22646659) Psychol Health Med. 2013.
5. Mulvaney SA, Anders S, Smith AK, Pittel EJ, Johnson KB., [A pilot test of a tailored mobile and web-based diabetes messaging system for adolescents.](http://www.ncbi.nlm.nih.gov/pubmed/22383802) J Telemed Telecare. 2012 Mar.
6. Hanauer DA, Wentzell K, Laffel N, Laffel LM. [Computerized Automated Reminder Diabetes System (CARDS): e-mail and SMS cell phone text messaging reminders to support diabetes management.](http://www.ncbi.nlm.nih.gov/pubmed/19848576)Diabetes Technol Ther. 2009 Feb.
7. Milovanovic I, Chantry M, Romon I, Druet C, Fagot-Campagna A, Levy-Marchal C., État de santé, scolarité et comportements à risque des adolescents diabétiques: l’étude Entred-Ado. Publiée sur le site de l'INVS.
8. [Benhamou PY](http://www.ncbi.nlm.nih.gov/pubmed?term=benhamou%20py%255bauthor%255d&cauthor=true&cauthor_uid=17395516)^1^, [Melki V](http://www.ncbi.nlm.nih.gov/pubmed?term=melki%20v%255bauthor%255d&cauthor=true&cauthor_uid=17395516), [Boizel R](http://www.ncbi.nlm.nih.gov/pubmed?term=boizel%20r%255bauthor%255d&cauthor=true&cauthor_uid=17395516), [Perreal F](http://www.ncbi.nlm.nih.gov/pubmed?term=perreal%20f%255bauthor%255d&cauthor=true&cauthor_uid=17395516), [Quesada JL](http://www.ncbi.nlm.nih.gov/pubmed?term=quesada%20jl%255bauthor%255d&cauthor=true&cauthor_uid=17395516), [Bessieres-Lacombe S](http://www.ncbi.nlm.nih.gov/pubmed?term=bessieres-lacombe%20s%255bauthor%255d&cauthor=true&cauthor_uid=17395516), [Bosson JL](http://www.ncbi.nlm.nih.gov/pubmed?term=bosson%20jl%255bauthor%255d&cauthor=true&cauthor_uid=17395516), [Halimi S](http://www.ncbi.nlm.nih.gov/pubmed?term=halimi%20s%255bauthor%255d&cauthor=true&cauthor_uid=17395516), [Hanaire H](http://www.ncbi.nlm.nih.gov/pubmed?term=hanaire%20h%255bauthor%255d&cauthor=true&cauthor_uid=17395516). One-year efficacy and safety of Web-based follow-up using cellular phone in type 1 diabetic patients under insulin pump therapy: the PumpNet study. [Diabetes Metab.](http://www.ncbi.nlm.nih.gov/pubmed/?term=benhamou+pumpnet+study) 2007 Jun;33(3):220-6. Epub 2007 Mar 28
9. [Rami B](http://www.ncbi.nlm.nih.gov/pubmed?term=rami%20b%255bauthor%255d&cauthor=true&cauthor_uid=16670859)^1^, [Popow C](http://www.ncbi.nlm.nih.gov/pubmed?term=popow%20c%255bauthor%255d&cauthor=true&cauthor_uid=16670859), [Horn W](http://www.ncbi.nlm.nih.gov/pubmed?term=horn%20w%255bauthor%255d&cauthor=true&cauthor_uid=16670859), [Waldhoer T](http://www.ncbi.nlm.nih.gov/pubmed?term=waldhoer%20t%255bauthor%255d&cauthor=true&cauthor_uid=16670859), [Schober E](http://www.ncbi.nlm.nih.gov/pubmed?term=schober%20e%255bauthor%255d&cauthor=true&cauthor_uid=16670859). Telemedical support to improve glycemic control in adolescents with type 1 diabetes mellitus. [Eur J Pediatr.](http://www.ncbi.nlm.nih.gov/pubmed/?term=rami+telemedical) 2006 Oct;165(10):701-5. Epub 2006 May 3.
10. Franklin VL, Waller A, Pagliari C, Greene SA. [A randomized controlled trial of Sweet Talk, a text-messaging system to support young people with diabetes.](http://www.ncbi.nlm.nih.gov/pubmed/17116184) Diabet Med. 2006 Dec;23(12):1332-8.
11. Charpentier G, Benhamou PY, Dardari D, Clergeot A, Franc S, Schaepelynck-Belicar P, Catargi B, Melki V, Chaillous L, Farret A, Bosson JL, Penfornis A; TeleDiab Study Group. [The Diabeo software enabling individualized insulin dose adjustments combined with telemedicine suppohttp://www.ncbi.nlm.nih.gov/pubmed/21266648rtimproves HbA1c in poorly controlled type 1 diabetic patients: a 6-month, randomized, open-label, parallel-group, multicenter trial (TeleDiab 1 Study).](http://www.ncbi.nlm.nih.gov/pubmed/21266648) Diabetes Care. 2011 Mar;34(3):533-9. doi: 10.2337/dc10-1259. Epub 2011 Jan 25.
12. Bin-Abbas B, Jabbari M, Al-Fares A, El-Dali A, Al-Orifi F. [Effect of mobile phone short text messages on glycaemic control in children with type 1 diabetes.](http://www.ncbi.nlm.nih.gov/pubmed/24643953) J Telemed Telecare. 2014;20(3):153-6. doi: 10.1177/1357633X14529244. Epub 2014 Mar 18.7
13. Mortensen HB, Hougaard P. [Comparison of metabolic control in a cross-sectional study of 2,873 children and adolescents with IDDM from 18 countries. The Hvidøre Study Group on Childhood Diabetes.](http://www.ncbi.nlm.nih.gov/pubmed/9135932) Diabetes Care. 1997 May;20(5):714-20. Erratum in: Diabetes Care 1997 Jul;20(7):1216.
14. Barat P, Lévy Marchal C. Epidemiology of diabetes mellitus in childhood. Arch Pediat. 2013 Dec;20 Suppl 4:S110-6. doi: 10.1016/S0929-693X(13)71424-6.
15. S. Malivoir. Impact de la dimension psychologique dans le traitement du diabète. L'estime de soi, frein ou moteur à la mise en place de stratégies de soins et d'adaptation à la maladie. Arch Pediatr 2013 Dec;20 Suppl 4:S144-8. doi: 10.1016/S0929-693X(13)71429-5.
16. N. Tubiana-Rufi. Les technologies au services de l'enfant atteint d'un diabète de type 1. Arch pediatr 2013 Dec;20 Suppl 4:S127-30. doi: 10.1016/S0929-693X(13)71426-X.
17. J. Beltrand, J.J. Robert. L'insulinothérapie en pédiatrie. Arch Pediat. 2013 Dec;20 Suppl 4
18. Mortensen HB, Robertson KJ, Aanstoot HJ et al, for the Hvidøre, Study Group on Childhood Diabetes : Insulin management and metabolic control of type 1 diabetes mellitus in childhood and

adolescence in 18 countries. Diabetic Med 1998 ; 15 : 752-9.

19. Holl RW, Swift PG, Mortensen HB, Lynggaard H, Hougaard P, Aanstoot HJ, Chiarelli F, Daneman D, Danne T, Dorchy H, Garandeau P, Greene S, Hoey HM, Kaprio EA, Kocova M, Martul P, Matsuura N, Robertson KJ, Schoenle EJ, Sovik O, Tsou RM, Vanelli M, Aman Insulin injection regimens and metabolic control in an international survey of adolescents with type 1 diabetes over 3 years: results from the Hvidore study group. J.Eur J Pediatr. 2003 Jan;162(1):22-9.

1. Zelen M. Randomized consent designs for clinical trials: an update. Stat Med. 1990 Jun;9(6):645-56.
2. [Hilliard ME](http://www.ncbi.nlm.nih.gov/pubmed?term=hilliard%20me%255bauthor%255d&cauthor=true&cauthor_uid=23340884)1, [Lawrence JM](http://www.ncbi.nlm.nih.gov/pubmed?term=lawrence%20jm%255bauthor%255d&cauthor=true&cauthor_uid=23340884), [Modi AC](http://www.ncbi.nlm.nih.gov/pubmed?term=modi%20ac%255bauthor%255d&cauthor=true&cauthor_uid=23340884), [Anderson A](http://www.ncbi.nlm.nih.gov/pubmed?term=anderson%20a%255bauthor%255d&cauthor=true&cauthor_uid=23340884), [Crume T](http://www.ncbi.nlm.nih.gov/pubmed?term=crume%20t%255bauthor%255d&cauthor=true&cauthor_uid=23340884), [Dolan LM](http://www.ncbi.nlm.nih.gov/pubmed?term=dolan%20lm%255bauthor%255d&cauthor=true&cauthor_uid=23340884), [Merchant AT](http://www.ncbi.nlm.nih.gov/pubmed?term=merchant%20at%255bauthor%255d&cauthor=true&cauthor_uid=23340884), [Yi-Frazier JP](http://www.ncbi.nlm.nih.gov/pubmed?term=yi-frazier%20jp%255bauthor%255d&cauthor=true&cauthor_uid=23340884), [Hood KK](http://www.ncbi.nlm.nih.gov/pubmed?term=hood%20kk%255bauthor%255d&cauthor=true&cauthor_uid=23340884); [SEARCH for Diabetes in Youth Study Group](http://www.ncbi.nlm.nih.gov/pubmed?term=search%20for%20diabetes%20in%20youth%20study%20group%255bcorporate%20author%255d). Identification of minimal clinically important difference scores of the PedsQL in children, adolescents, and young adults with type 1 and type 2 diabetes. [Diabetes Care.](http://www.ncbi.nlm.nih.gov/pubmed/23340884) 2013 Jul;36(7):1891-7. doi: 10.2337/dc12-1708. Epub 2013 Jan
3. [Varni JW](http://www.ncbi.nlm.nih.gov/pubmed?term=varni%20jw%255bauthor%255d&cauthor=true&cauthor_uid=12610013)1, [Burwinkle TM](http://www.ncbi.nlm.nih.gov/pubmed?term=burwinkle%20tm%255bauthor%255d&cauthor=true&cauthor_uid=12610013), [Jacobs JR](http://www.ncbi.nlm.nih.gov/pubmed?term=jacobs%20jr%255bauthor%255d&cauthor=true&cauthor_uid=12610013), [Gottschalk M](http://www.ncbi.nlm.nih.gov/pubmed?term=gottschalk%20m%255bauthor%255d&cauthor=true&cauthor_uid=12610013), [Kaufman F](http://www.ncbi.nlm.nih.gov/pubmed?term=kaufman%20f%255bauthor%255d&cauthor=true&cauthor_uid=12610013), [Jones KL](http://www.ncbi.nlm.nih.gov/pubmed?term=jones%20kl%255bauthor%255d&cauthor=true&cauthor_uid=12610013). The PedsQL in type 1 and type 2 diabetes: reliability and validity of the Pediatric Quality of Life Inventory Generic Core Scales and type 1 Diabetes Module. [Diabetes Care.](http://www.ncbi.nlm.nih.gov/pubmed/12610013) 2003 Mar;26(3):631-7.
4. S. Tessier, A. Vuillemin, J.-L. Lemelle, S. Briançon. [Propriétés psychométriques du questionnaire générique français «Pediatric Quality of Life Inventory Version 4.0](http://www.sciencedirect.com/science/article/pii/s1162908809000395). » Revue Européenne de Psychologie Appliquée/European Review of Applied Psychology, Volume 59, Issue 4, Pages 291-300
5. Vervloet M, van Dijk L, Santen-Reestman J, van Vlijmen B, van Wingerden P, Bouvy ML, de Bakker DH [SMS reminders improve adherence to oral medication in type 2 diabetes patients who are real time electronically monitored.](http://www.ncbi.nlm.nih.gov/pubmed/22652012). Int J Med Inform. 2012 Sep;81(9):594-604. doi: 10.1016/j.ijmedinf.2012.05.005. Epub 2012 May 30.
6. Chen SH, Tsai YF, Sun CY, Wu IW, Lee CC, Wu MS. [The impact of self-management support on the progression of chronic kidney disease--a prospective randomized controlled trial.](http://www.ncbi.nlm.nih.gov/pubmed/21414969) Nephrol Dial Transplant. 2011 Nov;26(11):3560-6. doi: 10.1093/ndt/gfr047. Epub 2011 Mar 17.
7. Haug S, Schaub MP, Venzin V, Meyer C, John U, Gmel G. [A pre-post study on the appropriateness and effectiveness of a Web- and text messaging-based intervention to reduce problem drinking in emerging adults.](http://www.ncbi.nlm.nih.gov/pubmed/23999406) J Med Internet Res. 2013 Sep 2;15(9):e196. doi: 10.2196/jmir.2755.
8. Nguyen B, Shrewsbury VA, O'Connor J, Steinbeck KS, Lee A, Hill AJ, Shah S, Kohn MR, Torvaldsen S, Baur LA. [Twelve-month outcomes of the loozit randomized controlled trial: a community-based healthy lifestyle program for overweight and obese adolescents.](http://www.ncbi.nlm.nih.gov/pubmed/22312175) Arch Pediatr Adolesc Med. 2012 Feb;166(2):170-7. doi: 10.1001/archpediatrics.2011.841.
9. Gentles SJ, Lokker C, McKibbon KA.[Health information technology to facilitate communication involving health care providers, caregivers, and pediatric patients: a scoping review.](http://www.ncbi.nlm.nih.gov/pubmed/20562092) J Med Internet Res. 2010 Jun 18;12(2):e22. doi: 10.2196/jmir.1390. Review.

29 Borus JS, Laffel L. [Adherence challenges in the management of type 1 diabetes in adolescents: prevention and intervention.](http://www.ncbi.nlm.nih.gov/pubmed/20489639) Curr Opin Pediatr. 2010 Aug;22(4):405-11. doi: 10.1097/MOP.0b013e32833a46a7. Review.

Appendix 2: Investigators and associated teams

|  | **Coordinating Investigator** | **Scientific Director** | **Cohort administrator** |
| --- | --- | --- | --- |
| **Title, first name, surname** | Professor Michel Polak | Professor Jean-Marc Treluyer | Nour Ibrahim |
| **Hospital** | Necker | Necker | Necker |
| **Department** | Paediatric Endocrinology | Paris Centre Clinical  Research Unit | Paris Centre Clinical  Research Unit |
| **Address** | 149 rue de Sèvres  75015 Paris | 149 rue de Sèvres  75015 Paris | 149 rue de Sèvres  75015 Paris |
| **Telephone** | +33 (0)1 44 49 48 02 | +33 (0)1 58 41 11 80 |  |
| **Fax** | +33 (0)1 44 38 16 48 | +33 (0)1 44 49 48 20 |  |
| **Email** | michel.polak@nck.aphp.fr | jean-marc.treluyer@parisdescartes.fr | nour.ibrahim@etu.parisdescartes.fr |

Appendix 3: INCLUSION DATA COLLECTION FORM

Impact of Mobile Phone Short Text Messages on the Glycaemic Control of Adolescents With Poorly Controlled Type 1 Diabetes.

Appointment date: …/.../... (dd/mm/yyyy)

1) Study data: to be completed by the coordinator

Inclusion number:.........................................................................

Surname initials:...........................................................................

First name initials:.........................................................................

Patient code: |__|__|__| |__|__|__| |__| |__|

2) General information: to be completed by the clinical practitioner

Date of birth: …/.../... (dd/mm/yyyy)

Gender: Female Male

Date of type 1 diabetes diagnosis: …/... (mm/yyyy)

3) HbA1c level

Sampling date for first HbA1c level: …/.../... (dd/mm/yyyy)

Result: ….... %

4) Treatment regimen

Treatment regimen: insulin syringes insulin pens insulin pump

5) Additional information

Medical history other than diabetes:......................................................................................................

History of psychiatric disorders:.............................................................................................................

6) Distribution of the capillary blood glucose diary: yes no

Percentage of insulin injections recorded _ _%

7) If the patient is assigned to the text messaging arm:

- Adolescent’s mobile phone number (where the text messages will be sent): _ _ _ _ _ _ _ _

- Time at which the text messages will be sent:

Product name and injection times

…......................................... …:... …:... …:... (hh:mm)

…......................................... …:... …:... …:... (hh:mm)

…......................................... …:... …:... …:... (hh:mm)

Appendix 4: 3 MONTH FOLLOW-UP DATA COLLECTION FORM

Impact of Mobile Phone Short Text Messages on the Glycaemic Control of Adolescents With Poorly Controlled Type 1 Diabetes.

Appointment date: …/.../... (dd/mm/yyyy)

1) Study data: to be completed by the coordinator

Patient code: |__|__|__| |__|__|__| |__| |__|

2) HbA1c level

Sampling date for second HbA1c level: …/.../... (dd/mm/yyyy)

Result: ….... %

3) Adverse event to be notified, comments:

…....................................................................................................................................................................

…....................................................................................................................................................................

4) Collection of the capillary blood glucose diary: yes no

Percentage of insulin injections recorded _ _%

5) Distribution and collection of the quality of life questionnaire: yes no

6) Distribution of the second capillary blood glucose diary: yes no

7) If the patient has been randomised to the text messaging arm:

Did s/he send a “STOP” text message yes no

If “yes”, date on which this text message was sent: …/.../... (dd/mm/yyyy)

If this is the case, in a single sentence, indicate the main reason given by the adolescent: ……………………………………………………………………….

Appendix 5: 6 MONTH FOLLOW-UP DATA COLLECTION FORM

Impact of Mobile Phone Short Text Messages on the Glycaemic Control of Adolescents With Poorly Controlled Type 1 Diabetes.

Appointment date: …/.../... (dd/mm/yyyy)

1) Study data: to be completed by the coordinator

Patient code: |__|__|__| |__|__|__| |__| |__|

2) HbA1c level

Sampling date for third HbA1c level: …/.../... (dd/mm/yyyy)

Result: ….... %

3) Adverse event to be notified, comments:

…....................................................................................................................................................................

…....................................................................................................................................................................

4) Collection of the capillary blood glucose diary: yes no

Percentage of insulin injections recorded _ _%

5) Distribution and collection of the quality of life questionnaires: yes no

6) If the patient has been randomised to the text messaging arm:

Did s/he send a “STOP” text message yes no

If “yes”, date on which this text message was sent: …/.../... (dd/mm/yyyy)

If this is the case, in a single sentence, indicate the main reason given by the adolescent: …..…………….

Appendix 6 – BLOOD GLUCOSE MONITORING DIARY

**Blood glucose
on waking**

**Insulin**

**Blood glucose after breakfast**

**Insulin**

**Blood glucose before lunch**

**Blood glucose after lunch**

**Blood glucose before dinner**

**Insulin**

**Blood glucose after dinner**

**Insulin**

**Blood glucose at bedtime**

**Comments**

Monday

Tuesday

Wednesday

Thursday

Friday

Saturday

Sunday

My blood glucose monitoring diary

Week …….. to ………..

**MY TARGET BLOOD GLUCOSE LEVEL
(to be defined with your doctor):**

**Weight:**


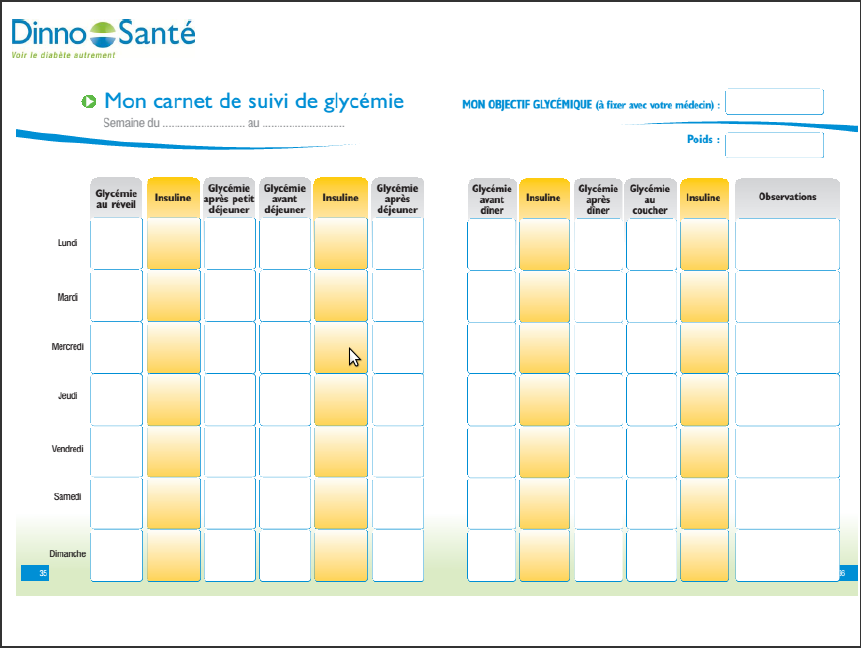


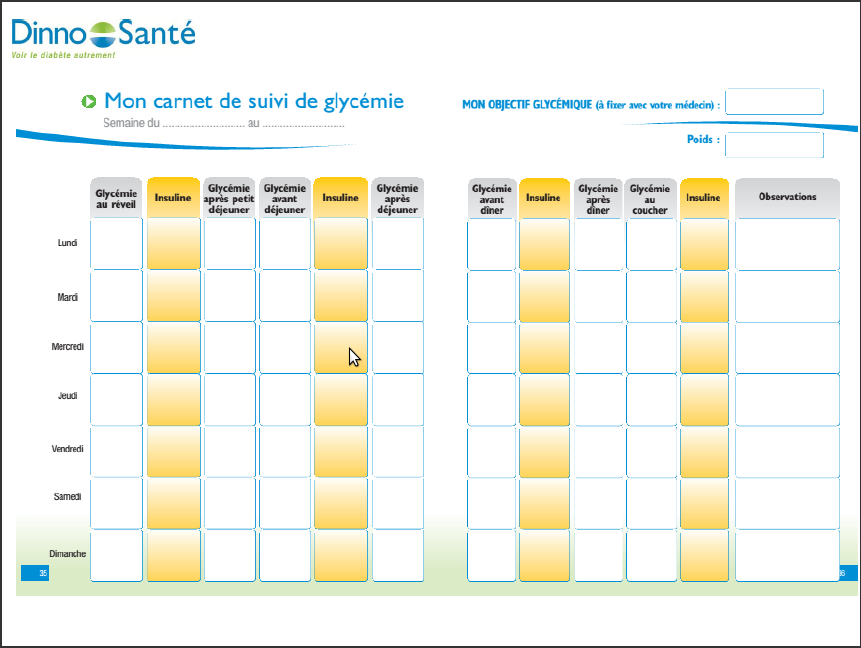


Appendix 7 – PARENT INFORMATION LEAFLET – PARTICIPATION IN A COHORT

Dear Sir/Madam,

The doctor responsible for your child’s treatment, Dr………………………….(surname, first name), practising at Hôpital …………………………, has invited your child to take part in a study on the treatment of his/her diabetes.

Please read through this information leaflet carefully as you may find answers to any questions you may think of before authorising your child to take part in this study.

You may contact the doctor responsible for your child’s care at any time during the study if you have any further questions.

1) What is the purpose of this study?

Your child has type 1 diabetes, an autoimmune disease which affects children and adolescents. The only currently available treatment for this disease is insulin, administered in several injections each day.

This treatment is very inconvenient. Hence, many adolescents find it difficult to follow their treatment regimen in a satisfactory manner.

This study aims to monitor the changes in HbA1c levels over the next 6 months. 100 young people between the ages of 12 and 21 years, with inadequately controlled type 1 diabetes, are expected to be included in this study.

2) What are the expected benefits?

By taking part in this study, your child will benefit from optimum management, allowing improved control of his/her diabetes. You will not incur any additional expenses. Furthermore, you will help us to gain greater insight into the management of type 1 diabetes among adolescents.

3) What are the foreseeable risks and constraints?

This study does not have any foreseeable risks.

The only expected constraint is having to attend an appointment three times for the study: an inclusion visit, and a visit after 3 months and 6 months of follow-up.

4) How long will your child be expected to participate in the study?

The planned study duration is 18 months, and your child will be expected to take part in the study for 6 months.

5) What is the study schedule?

During this study, your child will receive his/her usual medical care from his/her usual doctor, with:

- a medical appointment at inclusion: standard medical appointment (patient interview, medical examination), distribution of a capillary blood glucose diary, assay of HbA1c level
- a medical appointment at 3 months: standard medical appointment (patient interview, medical examination), assay of HbA1c level; together with collection of the first capillary blood glucose diary and distribution of a second capillary blood glucose diary; your child will also be asked to complete a quality of life questionnaire
- a medical appointment at 6 months: standard medical appointment (patient interview, medical examination), assay of HbA1c level; together with collection of the capillary blood glucose diary; your child will also be asked to complete a quality of life questionnaire

6) What are your rights as the parent of a child taking part in this study?

You may withdraw your child from this study at any time, without having to give a reason, without your decision compromising his/her continued treatment, quality of care or relationship with his/her doctor in any way. This will have no bearing on your child’s follow-up, which will be continued by the same medical team if you so wish.

7) Who should you contact if you have any questions or problems?

Study coordinator Contact details of the patient’s attending physician:

Ibrahim, Nour. Surname, first name:

Address: Clinical Research Unit Hospital, department:

CHU de Necker - Paris Telephone number

Parent surname(s) and first name(s).............................................................................................................

Minor’s surname and first name:....................................................................................................................

Date of patient information interview: __ __ / __ __ / __ __ __ __

Appendix 8 – INFORMATION LEAFLET FOR ADOLESCENTS AND YOUNG ADULTS – PARTICIPATION IN A COHORT

Dear Sir/Madam,

Your doctor, Dr………………………….(surname, first name), practising at Hôpital…………………….., has invited you to take part in a study on the changes in your diabetes.

Please read this information leaflet carefully as you may find answers to any questions you may have.

You may contact your doctor at any time during the study if you have any further questions.

1) What is the purpose of this study?

You have type 1 diabetes, an autoimmune disease which affects children and adolescents. The only currently available treatment to control this disease is insulin, administered in several injections each day. This treatment regimen is very difficult to follow. Hence, many adolescents and young adults find it difficult to follow their treatment regimen in a satisfactory manner.

This study aims to monitor the changes in HbA1c levels over the next 6 months. 100 young people between the ages of 12 and 21 years, with inadequately controlled type 1 diabetes, are expected to be included in this study.

2) What are the expected benefits?

By taking part in this study, you will benefit from optimum management, allowing improved control of your diabetes. Furthermore, you will help us to gain greater insight into the management of type 1 diabetes among adolescents and young adults.

3) What are the foreseeable risks and constraints?

This study does not have any foreseeable risks.

If you take part in this study, you will be required to attend three appointments: at inclusion, and after 3 months and 6 months of follow-up.

4) How long will you be expected to take part in this study?

You will take part in this study for a period of 6 months.

5) What is the study schedule?

During this study, you will receive standard medical care provided by your usual doctor, together with a quality of life questionnaire to be completed in 3 months, then in 6 months.

6) What are your rights as a patient taking part in this study?

You may withdraw from this study at any time, without having to give a reason. This will have no impact on your usual medical follow-up.

7) Who should you contact if you have any questions or problems?

Study coordinator Contact details of the patient’s attending physician:

Ibrahim, Nour. Surname, first name:

Address: Clinical Research Unit Hospital, department:

CHU de Necker - Paris Telephone number

Appendix 9 – PARENT INFORMATION LEAFLET - PARTICIPATION IN THE “TEXT MESSAGING” INTERVENTION

Dear Sir/Madam,

The doctor responsible for your child’s treatment, Dr…………………………(surname, first name), practising at Hôpital……………………… has invited your child to take part in a study on the treatment of his/her type 1 diabetes. It is important that you read through this leaflet carefully before making your decision. Please do not hesitate to ask for any further explanations.

1) What is the purpose of this study?

Your child is taking part in a cohort study on the management of changes in his/her diabetes. S/he has been randomly selected to receive reminder text messages. This study aims to test the hypothesis according to which text messaging support may improve the management of adolescents with inadequately controlled type 1 diabetes.

In response to the question raised, out of the 100 patients with inadequately controlled diabetes included in the cohort study, 50 will be included in the study aiming to assess the therapeutic efficacy of text messaging intervention.

2) What is the text message content, and when will the adolescent receive the text messages?

- Number of text messages:

Your child will receive several text messages each day, reminding him/her to administer his/her insulin injections. No text messages will relate to capillary blood glucose levels.

- Text messaging times:

Text messages will be sent at the usual insulin injection times based on your child’s personal therapeutic regimen. Hence, these times will be agreed with you during the study inclusion visit.

- Text message content:

The content of the messages will be as follows, for example:

“Remember to administer your slow-acting insulin injection.”

“You are about to have your meal; remember to administer your rapid-acting insulin injection.”

“Remember to administer your mixed insulin injection.”

- Confidentiality:

Your child’s mobile phone number will be kept confidential throughout the duration of the study, and will be destroyed once the study has ended.

3) What are the expected benefits?

The main expected benefit is improved treatment compliance, hence improved control of diabetes.

4) What are the foreseeable risks and constraints?

This study does not have any foreseeable risks. The only constraint in addition to participation in the cohort is that your child will receive daily text messages.

5) How long will your child be expected to participate in the study?

The planned study duration is 18 months, and your child will be expected to take part in the study for 6 months.

6) What is the study schedule?

During this study, your child will receive his/her usual medical care from his/her usual doctor, with:

- a medical appointment at inclusion: standard medical appointment (patient interview, medical examination), distribution of a capillary blood glucose diary, assay of HbA1c level
- a medical appointment at 3 months: standard medical appointment (patient interview, medical examination), assay of HbA1c level; together with collection of the first capillary blood glucose diary and distribution of a second capillary blood glucose diary; your child will also be asked to complete a quality of life questionnaire
- a medical appointment at 6 months: standard medical appointment (patient interview, medical examination), assay of HbA1c level; together with collection of the capillary blood glucose diary; your child will also be asked to complete a quality of life questionnaire.

7) What are your rights as the parents of a child taking part in this study?

You may withdraw your child from this study at any time, without having to give a reason, without your decision compromising his/her continued treatment, quality of care or relationship with his/her doctor in any way. This will have no bearing on your child’s follow-up, which will be continued by the same medical team if you so wish.

9) Who should you contact if you have any questions or problems?

Study coordinator Contact details of the patient’s attending physician:

Ibrahim, Nour. Surname, first name:

Address: Clinical Research Unit Hospital, department:

CHU de Necker - Paris Telephone number

Parents’ surname(s) and first name(s):

Minor’s surname and first name:

Date of patient information interview: __ __ / __ __ / __ __ __ __

APPENDIX 10 – INFORMATION LEAFLET FOR ADOLESCENTS AND YOUNG ADULTS - PARTICIPATION IN THE “TEXT MESSAGING” INTERVENTION

Dear Sir/Madam,

Your doctor, Dr ………………………… (surname, first name), practising at Hôpital………………… has invited you to take part in a study on your condition.

It is important that you read through this leaflet carefully before making your decision. Please do not hesitate to ask for any further explanations.

1) What is the purpose of this study?

You are taking part in a cohort study on the management of changes in your diabetes. You have been randomly selected to receive reminder text messages.

We would like to test the hypothesis according to which text messaging support may improve the management of adolescents and young adults with inadequately controlled type 1 diabetes.

In response to the question raised, out of the 100 patients with inadequately controlled diabetes included in the cohort study, 50 will be included in the study aiming to assess the therapeutic efficacy of text messaging intervention.

2) What is the text message content, and when will you receive the text messages?

- Number of text messages:

You will receive several text messages each day, reminding you to administer your insulin injections. No text messages will relate to capillary blood glucose levels.

- Text messaging times:

Text messages will be sent at the usual insulin injection times based on your personal therapeutic regimen. Hence, these times will be agreed with you during the study inclusion visit.

- Text message content:

The content of the messages will be as follows, for example:

“Remember to administer your slow-acting insulin injection.”

“You are about to have your meal; remember to administer your rapid-acting insulin injection.”

“Remember to administer your mixed insulin injection.”

- Confidentiality:

Your mobile phone number will be kept confidential throughout the duration of the study, and will be destroyed once the study has ended.

3) What are the expected benefits?

The expected benefit is improved control of your diabetes.

4) What are the foreseeable risks and constraints?

This study does not have any foreseeable risks. The only constraint is that you will receive several daily text messages.

5) How long will you be expected to take part in this study?

The planned study duration is 18 months, and you will be expected to take part in the study for 6 months.

6) What is the study schedule?

During this study, you will receive standard medical care provided by the doctor responsible for your follow-up, together with a quality of life questionnaire to be completed in 3 months, then in 6 months.

7) What are your rights as a participant in this study?

You may withdraw from this study at any time, without having to give a reason. This will have no impact on your usual medical follow-up.

8) Who should you contact if you have any questions or problems?

Study coordinator Contact details of the patient’s attending physician:

Ibrahim, Nour. Surname, first name:

Address: Clinical Research Unit Hospital, department:

CHU de Necker - Paris Telephone number

APPENDIX 11 – SATISFACTION SURVEY FOR ADOLESCENTS AND YOUNG ADULTS RANDOMISED TO THE TEXT MESSAGING ARM AFTER 6 MONTHS OF FOLLOW-UP.

You have been receiving text messages for 6 months as part of this study. Thank you for your participation.

Please could you now tell us what you think of this system (the support text messages you received) as we are interested in your opinion. Simply circle the answer which seems the most appropriate.

1. Did you find this system helpful?

No Not really Perhaps Yes

2. Did you find this system to be generally inconvenient?

No Not really Perhaps Yes

3. Did you find this system to be particularly inconvenient at weekends?

No Not really Perhaps Yes

4. Did you find the text messaging times convenient?

No Not really Perhaps Yes

5. Were there too many text messages?

No Not really I didn’t mind Perhaps Yes

6. Would you have preferred the text messages to be more personal?

No Not really I didn’t mind Perhaps Yes

7. If you had the choice, would you like to receive more of these text messages? Yes No

Thank you for completing this questionnaire.
